# VapC10 toxin of the legume symbiont *Sinorhizobium meliloti* targets tRNA^Ser^ and controls intracellular lifestyle

**DOI:** 10.1093/ismejo/wrae015

**Published:** 2024-01-29

**Authors:** Camille Syska, Aurélie Kiers, Corinne Rancurel, Marc Bailly-Bechet, Justine Lipuma, Geneviève Alloing, Isabelle Garcia, Laurence Dupont

**Affiliations:** Université Côte d'Azur, INRAE, CNRS, Institut Sophia Agrobiotech (ISA), Sophia Antipolis 06903, France; Université Côte d'Azur, INRAE, CNRS, Institut Sophia Agrobiotech (ISA), Sophia Antipolis 06903, France; Université Côte d'Azur, INRAE, CNRS, Institut Sophia Agrobiotech (ISA), Sophia Antipolis 06903, France; Université Côte d'Azur, INRAE, CNRS, Institut Sophia Agrobiotech (ISA), Sophia Antipolis 06903, France; Mycophyto, Grasse 06130, France; Université Côte d'Azur, INRAE, CNRS, Institut Sophia Agrobiotech (ISA), Sophia Antipolis 06903, France; Université Côte d'Azur, INRAE, CNRS, Institut Sophia Agrobiotech (ISA), Sophia Antipolis 06903, France; Université Côte d'Azur, INRAE, CNRS, Institut Sophia Agrobiotech (ISA), Sophia Antipolis 06903, France

**Keywords:** VapBC toxin–antitoxin, tRNAse, nitrogen-fixing symbiosis, bacteroid viability, root nodule senescence

## Abstract

The soil bacterium *Sinorhizobium meliloti* can establish a nitrogen-fixing symbiosis with the model legume *Medicago truncatula*. The rhizobia induce the formation of a specialized root organ called nodule, where they differentiate into bacteroids and reduce atmospheric nitrogen into ammonia. Little is known on the mechanisms involved in nodule senescence onset and in bacteroid survival inside the infected plant cells. Although toxin-antitoxin (TA) systems have been shown to promote intracellular survival within host cells in human pathogenic bacteria, their role in symbiotic bacteria was rarely investigated. *S. meliloti* encodes several TA systems, mainly of the VapBC family. Here we present the functional characterization, through a multidisciplinary approach, of the VapBC10 TA system of *S. meliloti*. Following a mapping by overexpression of an RNase in *Escherichia coli* (MORE) RNA-seq analysis, we demonstrated that the VapC10 toxin is an RNase that cleaves the anticodon loop of two tRNA^Ser^. Thereafter, a bioinformatics approach was used to predict VapC10 targets in bacteroids. This analysis suggests that toxin activation triggers a specific proteome reprogramming that could limit nitrogen fixation capability and viability of bacteroids. Accordingly, a *vapC10* mutant induces a delayed senescence in nodules, associated to an enhanced bacteroid survival. VapBC10 TA system could contribute to *S. meliloti* adaptation to symbiotic lifestyle, in response to plant nitrogen status.

## Introduction

*Sinorhizobium meliloti* is a soil bacterium found either in a free-living state in the rhizosphere, or as intracellular, nitrogen (N_2_)-fixing microsymbiont of legumes (*Fabaceae*) such as *Medicago truncatula* and *Medicago sativa* (alfalfa)*.* In this symbiotic interaction, bacteria induce the formation of a new organ, the root nodule. Within this nodule, the bacteria, differentiated in bacteroids, reduce atmospheric nitrogen to ammonia through the activity of nitrogenase. This bioavailable nitrogen source is used by the host plant to fulfill its nutrient demand and, in exchange, the bacteroids are provided in carbon and energy with C_4_-dicarboxylates produced through photosynthesis [1].

The symbiotic interaction between *S. meliloti* and *M. truncatula* results in the formation of indeterminate nodules containing four distinct zones [2-4]. The root distal meristematic zone (ZI) is responsible for the continuous elongation of the nodule. In the infection zone (ZII), dividing bacteria progress through an infection thread and are released by endocytosis into the cytoplasm of submeristematic plant cells to form a symbiosome. This new entity shortly divides before bacteria irreversibly differentiate into growth-arrested nitrogen-fixing bacteroids. This process involves global metabolic reprogramming of the microsymbiont, modifications of the bacteroid membrane structure, and cell size enlargement, associated to genome endoreduplication [5, 6]. In the nitrogen-fixing zone (ZIII), the bacteroids have a metabolism entirely devoted to nitrogen fixation, realized in microoxic conditions [1]. Five to six weeks post-inoculation (wpi), the root proximal zone of nodule (ZIV) displays senescence, and the bacteroids degrade and collapse, leading to decreased nitrogen fixation [7, 8].

Little information exists on the mechanisms involved in the survival of *S. meliloti* inside root nodule-infected cells. In various host–pathogen interactions, the adaptation of bacteria to intracellular lifestyle has been shown to involve toxin-antitoxin (TA) systems [9]. TA systems are found in nearly all bacteria and archaea and are encoded by genes organized in operons [10]. They are composed of a toxin which targets specific cellular functions and an antitoxin that counteracts the action of the toxin. TA modules are classified into eight different types (I–VIII) according to the mechanism of toxin neutralization by the antitoxin [11-13]. Type II TA systems are the best characterized [14, 15] and the most abundant, especially in bacterial species which are exposed to diverse microenvironments [16, 17]. They are classified in eight superfamilies depending on the structure and activity of the toxin. The VapBC superfamily is the most preponderant in bacterial genomes [18, 19]. In human pathogens, the VapBC modules contribute to virulence and bacterial survival during infection [9, 13, 16, 20-22]. The VapC toxin is a site-specific ribosome-independent PilT N‐terminus (PIN) endoribonuclease mainly identified as a tRNase and containing three strictly conserved acidic residues in enzymatic active site [23, 24]. Its activity is blocked by protein–protein interaction with its cognate antitoxin VapB. The TA complex exerts a negative autoregulation on the expression of *vapBC* genes by binding to the operator site of the TA promoter [25, 26]. Under stress conditions, the unstable antitoxin is degraded by endogenous proteases [27], leading to the deregulation of the TA operon and synthesis of an active toxin [27-29].

The chromosome of *S. meliloti* carries 29 operons that potentially encode Type II TA systems, according to the Toxin-Antitoxin Database (TADB 2.0) [19]. Eleven belong to the VapBC family, VapBC1 to VapBC7 identified by Pandey and Gerdes [18], and four modules identified later (TADB 2.0) that we named VapBC8 to VapBC11. Among them, NtrPR (VapBC4) and VapBC5 have been shown to control the efficiency of symbiosis with alfalfa (*M. sativa*) [30-32]. Inactivation of the toxin results in an improved nitrogen fixation capacity of both *vapC* mutants, suggesting that these TA modules may influence bacteroid metabolism. However, their RNA targets and their link with the symbiotic phenotype of the *vapC* mutants were not investigated.

Here, we show that the VapBC10 module of *S. meliloti* acts as a TA system and that VapC10 cleaves the anticodon loop of two specific tRNA^Ser^. An *in silico* analysis and predictive modelling tools of VapC10 targets in nodules suggest that VapC10 affects the synthesis of several symbiotic proteins, some necessary for the synthesis and functioning of nitrogenase. *M. truncatula* plants inoculated with a *vapC10* mutant form root nodules with increased nitrogen fixation efficiency, associated with a higher intracellular viability of bacteroids compared to wild-type (WT)-induced nodules. The VapBC10 module may control bacteroid adaptation, possibly in response to host plant nitrogen demand.

## Materials and methods

### Bacterial strains and growth conditions

Strains used in this study are listed in Table S1. *Escherichia coli* strains were grown at 37°C in Luria-Bertani (LB) medium supplemented with 100 μg.ml^−1^ ampicillin, 30 μg.ml^−1^ kanamycin, 34 μg.ml^−1^ chloramphenicol, 0.2% glucose, 1% arabinose, or 100 μM isopropyl beta-D-thiogalactoside (IPTG), when appropriate. *S. meliloti* strains were grown at 30°C in LB medium supplemented with 2.5 mM MgSO_4_ and 2.5 mM CaCl_2_(LB_MC_), and appropriate antibiotics for the selection of strains (streptomycin 100 μg.ml^−1^ and neomycin 200 μg.ml^−1^).

### Construction of the *vapB10* and *vapC10* expression plasmids

Using appropriate primers (Table S2), the *vapC10* and *vapB10* coding regions of the WT strain 2011 of *S. meliloti* were first cloned into the pJET1.2 vector (ThermoFisher), resulting in pJET-*vapC10-tox* and pJET-*vapB10-tox* plasmids, respectively. Then, the BsaI-SalI fragment of pJET-*vapC10-tox* plasmid was subcloned into the NcoI/SalI sites of pBAD24 [33], giving pBAD24-*vapC10* plasmid. The BsaI-XhoI fragment of pJET-*vapB10-tox* plasmid was subcloned into the NcoI/SalI sites of pRSF1b (Novagen), giving pRSF1b-*vapB10* plasmid (Table S1).

### Construction of the *S. meliloti vapC10* mutant

An internal fragment of *vapC10* gene from strain 2011 of *S. meliloti*, obtained by polymerase chain reaction (PCR) amplification using appropriate primers (Table S2), was first inserted into the pJET1.2 vector, before subcloning into the suicide vector pK19mob2ΩHMB using the HindIII and BsrGI restriction enzymes. The resulting plasmid pK19mob2ΩHMB-*vapC10* (Table S1) was introduced into *S. meliloti* strain 2011 by triparental mating [34]. The *vapC10* mutant was selected by a simple recombination event resulting in the disruption of the coding sequence of the toxin gene in the last 183 bp on 444 bp. The disruption of the *vapC10* gene was verified by PCR amplifications.

### Toxin and antitoxin activity of VapBC10 TA system

Two independent cultures of *E. coli* DH5α carrying pBAD24-*vapC10* or pBAD24 were grown overnight at 37°C in LB supplemented with ampicillin and 0.2% glucose. Then, each culture was split in two, diluted to an OD_600nm_ = 0.05 and grown to an OD_600nm_ = 0.4. The *vapC10* expression was induced in one culture by adding 1% of L-arabinose and repressed in the other culture by 0.2% glucose. At chosen time points, 10 μl of several serial dilutions were plated on LB agar supplemented with glucose in technical duplicates. After 16 h of incubation at 37°C, the number of viable bacteria (colony-forming unit (CFU)) was determined by the average of the two spots of each independent culture. The putative VapB10 antidote effect was tested in *E. coli* BL21 (DE3) pLysS carrying pBAD24-*vapC10* or pRSF1b-*vapB10* or both. Strains were grown overnight in LB containing glucose and appropriate antibiotics. Then, cells were diluted at OD_600nm_ = 0.05 and grown until OD_600nm_ = 0.15 in LB with appropriate antibiotics. To induce plasmid expression, L-arabinose (1%) and IPTG (100 μM) were added to the distinct cultures. Growth kinetics were followed at OD_600nm_ for 4 h. These toxic-antitoxic assays were realized in three independent biological replicates.

### Plant growth conditions

Seeds of *M. truncatula* ecotype A17 were surface sterilized and germinated as previously described [35]. Plants were grown in pots filled with sand B5 under a 16h light/8h dark photoperiod, associated with 23°C/21°C thermoperiod, and watered with nitrogen-free nutrient medium [36]. Plantlets were inoculated 6 days after germination with *S. meliloti* WT or *vapC10* mutant strains (10 ml per plant of resuspended bacteria in water at OD_600nm_ = 0.05). Plants and nodules were harvested 3- and 6-wpi corresponding, respectively, to the absence and onset of nodule senescence in WT-induced nodules.

### Nitrogen fixation capacity assay, symbiotic, and nodule phenotyping

Nitrogen fixation activity was determined by the acetylene reduction assay (ARA) as described previously [37]. Briefly, for each condition, roots of 15 plants (three plants per vial) were incubated for 30 min at 28°C with 10% (v/v) of acetylene. The reduction of acetylene to ethylene was analyzed by gas chromatography (Agilent). The activity of nitrogenase is expressed in nanomoles of ethylene produced per hour and per nodule, or per plant.

To quantify the proportion of senescent nodules, harboring a typical green color due to degraded leghemoglobin, nodules (6 wpi) induced by the WT or *vapC10* mutant strains were harvested 5 cm below the crown and observed with a Leica MZFLIII binocular magnifier. The nodule length and the relative size of the senescence zone were measured by using AxioVision LE software.

For the statistical analysis of the symbiotic parameters (N_2_ fixation capacity, weight of the aerial parts of the plant, number of nodules, and nodule senescence), the significance of differences was assessed using one-way analysis of variance (R software). The *P* values and tests used for each assay are detailed in Tables S3 and S4.

To determine the expression of plant senescent markers, nodules were harvested at 6 wpi, on the 5 cm below the crown. Nodule RNA extraction, DNase treatment, cDNA synthesis, reverse transcription quantitative PCR (RT-qPCR), and statistical analysis were performed as previously described [38]. The primers used for the RT-qPCR are listed in Table S2. The reference genes used for normalization were *MTC*27 and *A38*. The results presented are the means of three independent biological experiments.

To assess the bacteroid viability in nodules, an *in situ* Live/Dead staining was realized. Nodules harvested at 6 wpi were embedded in 6% (w/v) agarose. Sections (120 μm) were prepared with a Leica HM650V vibratome and incubated in Live/Dead staining fluorescent dyes (Live/Dead *Bac*Light kit, Invitrogen) as previously described [39]. Images were acquired with a Zeiss confocal laser scanning microscope (LSM880).

### Identification of the molecular targets of VapC10 toxin by mapping by overexpression of an RNase in *E. coli* (MORE) RNA-seq

#### Bacterial growth conditions and RNA extraction

**Figure 1:**
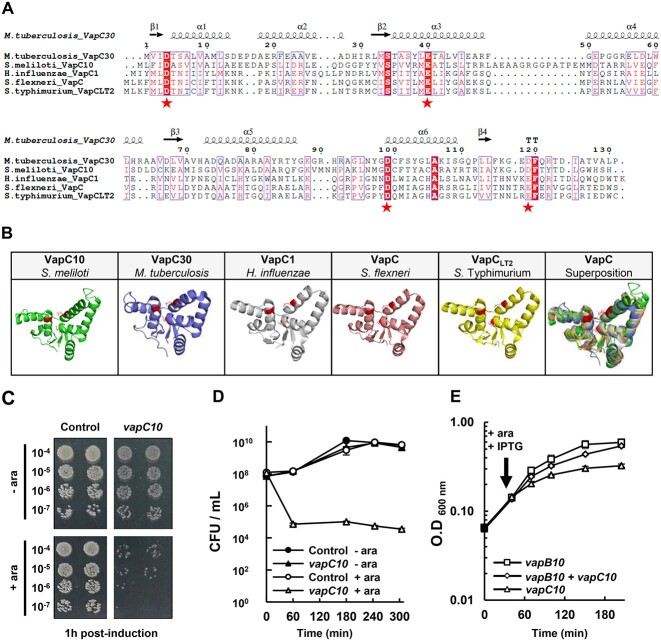
*vapBC10* operon encodes a TA system; (A) multiple sequence alignment of VapC10 toxin of *S. meliloti* with previously characterized VapC homologs in other bacteria; the VapC10 protein was compared to various PIN domain VapC toxins whose structure has been previously solved by crystallography; the sequence alignment was realized using PROMALS3D webserver [40]; the positions of the alpha helices (α) and beta sheets (β) deduced by PROMALS3D are indicated above the alignment; the four conserved acidic amino acid residues required for the catalytic activity of the PIN domain of VapC toxins are indicated by stars at the bottom of the alignment; (B) comparison of the 3D structure of VapC toxins; the structure of *S. meliloti* VapC10 was predicted using TrRosetta [41] and that of VapC homologs was extracted from Protein Data Bank (PDB accession: VapC30 of *Mycobacterium tuberculosis* “4XGQ” [42]; VapC1 of *H. influenzae* “6NKL” [43]; VapC of *S. flexneri* “3TND” [44]; VapC_LT2_ of *S.* Typhimurium “6IFC” [45]); all the 3D models were visualized using Pymol software; (C, D) toxicity assay of the VapC10 protein; *E. coli* DH5α was transformed with pBAD24 (Control) or pBAD24-*vapC10* (*vapC10)* plasmids (Table S1), and induced with 1% of arabinose (+ ara) or not (− ara); (C) colony-forming unit (CFU) per ml at 1 h postinduction; cells grown until OD_600nm_= 0.4 were induced by arabinose (T_0_) for 1 h, serially diluted as indicated, and spotted on LB glucose agar plates; (D) kinetics of viability (CFU/ml); (E) antitoxicity assay of VapB10 protein; *E. coli* BL21 (DE3) pLysS was transformed with pRSF1b-*vapB10* (IPTG inducible)*,* pBAD24-*vapC10* (arabinose inducible) or co-transformed with both plasmids; cells were grown until OD_600nm_ = 0.15, induced by arabinose and IPTG at the indicated time (arrow), and their growth capacity was followed by OD_600nm_ measurement for 3 h; results shown are a representative example of three independent biological replicates; the means of the technical duplicates from each condition are shown on the viability (1D) and growth (1E) curves.

The 5′ RNA-seq approach used to identify VaC10 cleavage sites was performed in *E. coli*, as tightly controlled expression systems are not available in *S. meliloti*. First, the plasmids pBAD24 and pBAD24-*vapC10* were introduced into the *E. coli* BW25113∆6 strain which is deleted in six Type II TA systems. Overnight cultures grown in LB with 0.2% glucose were diluted to OD_600nm_ = 0.05. When cultures reached OD_600nm_ = 0.4, the medium was supplemented with 1% of arabinose, and cells were grown for a further 30 min. As 30 min of induction slows down the bacterial growth (Fig. 1E), we chose this timing to be as stringent as possible for the mapping by overexpression of an RNase in *E. coli* (MORE)-RNAseq experiment. Then, total RNA extraction was performed as previously described [46], and 20 μg of RNA were treated with RQ1 RNase-Free DNase (Promega), following the manufacturer instructions. Then, RNAs were purified with a phenol:chloroform extraction. The quantity and quality of total RNA (Fig. S1) were estimated using a Bioanalyser Agilent 2100 (Prokaryote Total RNA nanochip).

#### Preparation of cDNA libraries for Illumina RNA-sequencing

As VapC toxins could produce either 5′-hydroxyl (5′-OH) or 5′-monophosphate (5′-P) moieties, both types of transcripts were selected for the 5′ RNA-seq experiments (Fig. S2). To convert 5′-OH into 5′-P ends, the libraries were treated with T4 polynucleotide kinase. Then, Illumina 5′-adaptors with a barcode were ligated to 5′-P ends of RNAs, discriminating the uncleaved RNAs having 5′-PP or 5′-PPP ends. After ligation, the generation of cDNA banks was performed by reverse transcription using a common 3′ Illumina adaptor. Only cDNAs that migrated below 300 nucleotides were isolated after gel excision. Finally, the cDNA libraries were sequenced by a 1 × 150 bp single-end Illumina MiSeq Nano chip (Fasteris sequencing platform, Switzerland).

#### Bioinformatic analysis: cleaning, mapping, and counting table of reads

After sequencing, the sequence reads were processed as follow. First, adaptors used for sequencing and sequence reads below 27 nucleotides were trimmed. Then, trimmed cDNA banks were subjected to quality filtering with FastQC prior mapping to the BW25113 genome (CP009273.1), by using bowtie2 (version 2.3.5) within the parameters very-sensitive -D 20 -R 3 -N 0 -L 20 -i S,1,0.50, end-to-end as described in Culviner *et al*. [47]. For each condition, between 7.5 and 9.6 million reads were aligned to the BW25113 genome (Table S5). Interconversion of Sequence Alignment Map (SAM) and Binary Alignment Map (BAM) file formats was conducted by using the SAMtools (version 1.7), with htslib 1.7 (Genome Research Ltd). The normalization of BAM files was performed within deepTools (version 3.1.3), tool = bamCoverage. Finally, alignments and coverage of each bank were observed on the portal “myGenomeBrowser.”

#### Statistical analysis of the MORE RNA-seq data

To identify the target of VapC10, a counting table was performed on the 5′-end reads. All the sequence reads, in the correct orientation, having at least one mismatch on the first 30 nucleotides, were removed before the mapping, as described in Schifano *et al.* [46]. Multi-mapped reads were counted as 1/x where x is the number of mapping targets in the genome. A “signature” of cleaving was investigated where, only in VapC10 condition, 5′-ends of reads are perfectly aligned at the same nucleotide position, whereas in the control condition, this signature is not observed. A script was elaborated on R to generate a counting table calculating a score, computed for each position *i* as follows:


\begin{align*}& Score\ \left(\mathrm{n}\right)i=\\&\frac{{\left( mediane\left( number\ of\ reads\ \right(R1\right)}_i+{(R2)}_i\left)\right)+0.5}{\sum_{k=i-1}^{i-10}{\left( mediane\ \left( number\ of\ reads\ \right(R1\right)}_k+{(R2)}_k\left)\right)+\left(10\ast 0.5\right)}\end{align*}

$$ Cleavage\ ratio\ i=\frac{Score\ \left(\mathrm{VapC}\right)i}{Score\ \left(\mathrm{Ctrl}\right)i} $$

This local peak score is only based on reads number at *i* position and positions located up to 10 nucleotides upstream. The 0.5 factors appearing mean that all read counts were incremented by 0.5 to prevent 0 counts. Then, the cleavage ratio at *i* position between VapC10 and Control is determined and fixed at >60. Analysis of the highest scores allowed to find among targets, three tRNA^Ser^. To identify tRNA^Ser^ homologs in *S. meliloti*, the sequences of these *E. coli* tRNA^Ser^ were then compared to the 55 tRNAs of *S. meliloti* using LocARNA, an alignment algorithm that accounts for the conservation of structural RNA features [48].

### *In silico* predictions of the VapC10 toxin activity on *S. meliloti* proteome in the fixation zone of the nodule

The impact of the cleavage of the tRNA^Ser(GGA)^ and tRNA^Ser(UGA)^ by VapC10 on *S. meliloti* proteome was investigated *in silico*. Both tRNAs SMc02955 and SMc01243 were supposed to be cleaved by VapC10 in *S. meliloti* bacteroids. We restricted the studied proteome to the translation of the 2209 genes specifically and significantly overexpressed in the fixation zone among 8933 genes. In this zone, the distribution of the protein length and the codon frequency of the six serine codons were similar to the full proteome. To limit size biases, absolute numbers of codons were used rather than proportions of specific codons. Thus, groups of genes of interest were computed, using different criteria (Fig. S3). “ZIII+” list contains genes with an expression above 1500 reads. “Rare *serX/SMc02915*” list contains RNAs with at least 4 UCU codons, whereas “*serX/SMc02915*” list also contains those having at least 12 UCC codons, both UCC and UCU being decoded by tRNA^Ser(GGA)^. A similar procedure was used to create a “*serV/SMc02409*” list, for codons AGU and AGC, both decoded by the tRNA^Ser(GCU)^. Finally, for tRNA^Ser(UGA)^ and tRNA^Ser(CGA)^, decoding a single codon, “*serT*/*SMc01243*” and “*serU*/*SMc03779*” respective lists were computed if transcripts contained at least 4 UCA codons or 12 UCG codons, respectively. Venn diagrams were realized from these different lists.

## Results

### *vapBC10* operon encodes a functional toxin-antitoxin system

The *vapBC10* genes are organized in an operon on the *S. meliloti* chromosome, the antitoxin gene *vapB10* (SMc02988) and the toxin gene *vapC10* (SMc02987) overlapping each other by one nucleotide (Fig. S4A). The *vapB10* gene encodes a protein of 8.9 kDa containing a helix-turn-helix (HTH) DNA binding domain that may allow the negative autoregulation of the operon transcription as previously described for other VapBC systems [10]. The VapB10 protein shares 44% sequence similarity with the VapB30 antitoxin of *Mycobacterium tuberculosis* (Fig. S4B). The *vapC10* gene encodes a putative PIN domain of 16.2 kDa sharing 43% sequence similarity with VapC30 of *M. tuberculosis* [42], and 25% with VapC_LT2_ of *Salmonella* Typhimurium [45], VapC of *Shigella flexneri* [44], and VapC1 of *Haemophilus influenzae* [43] (Fig. 1A). VapC10 contains the characteristic α/β/α sandwich structure of PIN domain, including at least the three acidic amino acids essential for catalysis (Fig. 1A and B). Besides, the VapC10 protein presents an extension of 15 aa residues that could form an extra loop between helixes 3 and 4 (Fig. 1B).

To determine whether VapBC10 acts as a TA module, the ability of VapC10 to affect bacterial viability was first assessed, by using an *E. coli* strain carrying the *vapC10* gene under the control of an arabinose-inducible promoter (Fig. 1C and D). A 10^3^-fold decrease of CFU/ml was observed 1 h after the addition of arabinose in the culture of bacteria expressing *vapC10* (Fig. 1D and Fig. S5A and B), showing the strong toxicity of the VapC10 protein.

The ability of the antitoxin VapB10 to counteract the effect of VapC10 was thereafter tested by monitoring the growth of bacteria carrying the *vapB10* and *vapC10* genes under the control of IPTG and arabinose-inducible promoters, respectively. Fig. 1E and Fig. S5C and D show that the addition of inducers inhibited the growth of bacteria expressing VapC10 after 30 min, and stopped it in <2 h. In contrast, no growth defect was observed in bacteria expressing both VapC10 and VapB10. Thus, VapB10 acts as a VapC10 antidote.

Taken together, these results demonstrate that the *vapBC10* operon encodes a functional TA system.

### VapC10 toxin cleaves the anticodon loop of two tRNA^Ser^ in *E. coli*

**Figure 2:**
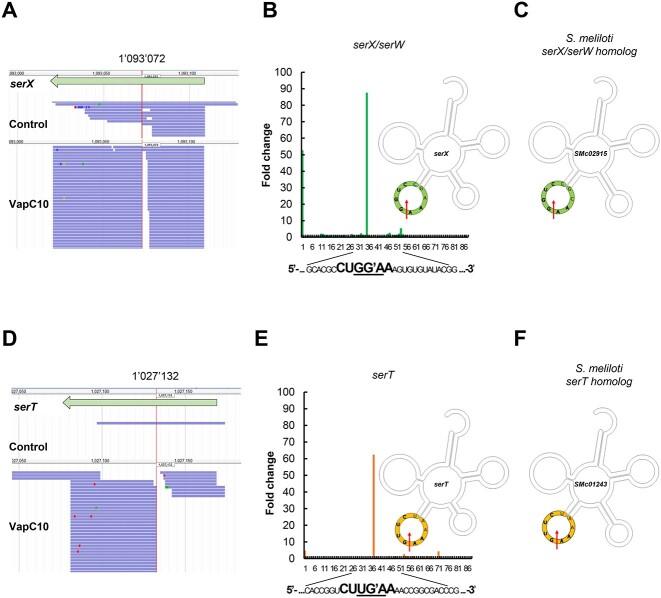
VapC10 cleaves tRNA^Ser(GGA)^ and tRNA^Ser(UGA)^ in the CU(U/G)G^AA anticodon loop in *E. coli*; (A, D) mapping of sequencing reads identified using the MORE RNA-seq method on *E. coli* genome; examples correspond to one replicate obtained on the *serX* (A) and the *serT* (D) tRNAs; genes are represented by arrows and reads by lines; color dots in the reads represent punctual nucleotide differences; the 5′ RNA moieties generated by VapC10 cleavage correspond to the 1 093 072-nucleotide position of *serX* gene (A) and the 1 027 132 nucleotide position of *serT* gene (D) on the *E. coli* BW25113∆6 genome (vertical lines); (B, E) histograms representing the fold change of sequencing reads between the induced and noninduced VapC10 conditions, in the tRNA^Ser(GGA)^ (*serX* and *serW*) (B) and in the tRNA^Ser(UGA)^ (*serT*) (E), at each nucleotide position; the cleavage site is shown in bold letters in the tRNA sequences, and the anticodon is underlined; the specific position of VapC10 cleavage in the anticodon loop is shown in the secondary structure of the corresponding *E. coli* tRNAs; the numbers shown below the histograms indicate the nucleotide positions of *serX* and *serT* tRNAs; (C, F) predicted structures of tRNA^Ser^ and best homologs of *E. coli* tRNA^Ser^ in *S. meliloti*; the best homologs of the VapC10-targeted tRNAs, specified by *serX* and *serT* in *E. coli*, are SMc02915 and SMc01243, respectively; the corresponding specific position of VapC10 cleavage in the anticodon loop is shown by an arrow in the secondary structure of the corresponding tRNAs in *E. coli* and *S. meliloti*; the consensus cleavage site is shown in bold letters in the tRNA sequences.

VapC toxins are site-specific RNases described to target mainly tRNAs, and much more rarely mRNAs or rRNAs. To identify the RNA target(s) of VapC10, we used the MORE RNA-seq technique, adapted from a previously published method [46]. A cleavage site was considered as toxin specific when an enrichment of reads higher than 60 was observed between the VapC10 and control conditions. Seven VapC10 RNA targets were identified in *E. coli* (Fig. S6A). VapC10 cleaves specifically three tRNA^Ser^ i.e. the two tRNA^Ser(GGA)^ encoded by the identical genes *serX* and *serW* and the tRNA^Ser(UGA)^ transcribed by *serT* (Fig. 2A and D). The two other tRNA^Ser^ encoded by *serU* and *serV* are not targeted by VapC10 (Fig. S6D and E). From this analysis, a consensus sequence of the VapC10 cleavage site, CU(U/G)G^AA could be defined in the anticodon loop (Fig. 2B and E). VapC10 also specifically cleaves four mRNAs, *msrB*, *sdaA*, *msbA*, and *kbl*, at a G^AA site located in a potential loop structure (Fig. S6B). The enzyme may recognize, in these mRNAs, both sequence and RNA structure of *serX* and *serT* tRNA anticodon stem-loops. However, the homologs of *msrB*, *sdaA*, *msbA*, and *kbl* in *S. meliloti*, respectively, *msrB1*, *sda*, SMb20813, and SMc01565, cannot be physiological targets of VapC10 because they lack the minimal consensus cleavage site G^AA. Thus, further analysis was focused on the tRNAs targeted by VapC10 in *S. meliloti*.

Among the 55 genes encoding tRNAs in the *S. meliloti* genome, the homologs of *serX* and *serT* genes were identified by using the LocaRNA software, which accounts for both sequence and secondary structure similarity [48]. SMc02915, SMc01243, SMc03779, and SMc02409 genes of *S. meliloti* share the best similarity score with the *E. coli* genes *serX*, *serT*, *serU*, and *serV*, respectively (Fig. S6C). Moreover, the consensus sequence of VapC10 cleavage site is only present in the sequence of the tRNA^Ser^ encoded by SMc02915 and SMc01243 genes (Fig. 2C and F and Fig. S6C).

We performed a genetic approach to show that the *S. meliloti* tRNAs SMc01243 and SMc02915 were effectively cleaved by VapC10 in *E. coli*. The tRNA^Ser(UGA)^ SMc01243, tRNA^Ser(GGA)^ SMc02915, or tRNA^Ala(CGC)^ SMc01244 used as a negative control, were expressed in *E. coli* cells carrying *vapC10* gene under the control of the pBAD promoter. Our results show that the only expression of SMc01243 or SMc02915 counteracted the effect of VapC10 on bacterial growth (Fig. S7 and Supplementary Information). These findings demonstrate that the *S. meliloti* tRNA^Ser^ encoded by SMc01243 and SMc02915 are specifically cleaved by VapC10 toxin.

Taken together, these results demonstrate that VapC10 cleaves two tRNA^Ser^ in *E. coli* and recognizes the homologous tRNAs encoded by SMc02915 and SMc01243 in *S. meliloti*.

### VapC10 potentially affects NifA and FixC synthesis during symbiosis

**Figure 3:**
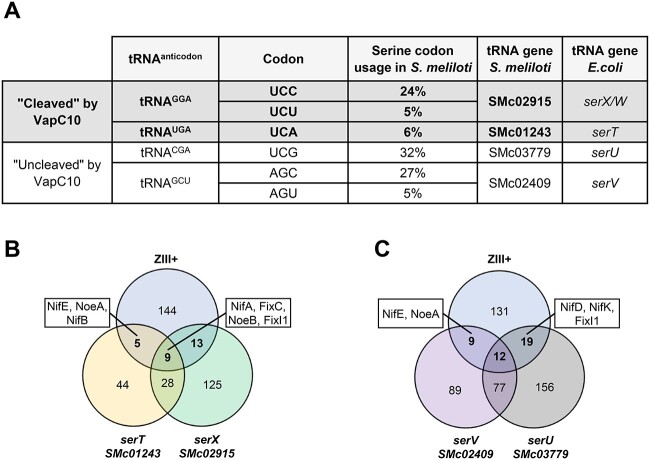
Transcripts of the fixation zone, rich in specific serine codons; (A) recognition of the six serine codons by the four tRNA^Ser^ and their relative codon usage in *S. meliloti*; (B) transcripts rich in serine codons translated by tRNA^Ser(UGA)^ and tRNA^Ser(GGA)^, predicted to be impacted by VapC10 cleavage; a Venn diagram was used to identify genes encoding mRNAs containing at least 4 rare UCA codon (named *serT/SMc01243* list), and mRNAs containing at least 12 frequent UCC codon or containing at least 4 rare UCU codon (named *serX/SMc02915* list), in the specifically and highly expressed genes (*n* ≥ 1500 reads) of the fixation Zone III (named ZIII+ list); (C) transcripts rich in serine codons translated by tRNA^Ser(GCU)^ and tRNA^Ser(CGA)^, predicted to be not impacted by VapC10 cleavage; a Venn diagram was used to identify genes encoding mRNAs containing at least 12 frequent AGC codon or containing at least 4 rare AGU codon (named *serV*/*SMc02409* list), and mRNAs containing at least 12 frequent UCG codon (named *serU*/*SMc03779* list), in the specifically and highly expressed genes (*n* ≥ 1500 reads) of the fixation Zone III (named ZIII+ list); lists of genes are shown in File S1; FixC, oxidoreductase involved in nitrogenase functioning; FixI1, cation transporter ATPase subunit; NifA, transcriptional activator of nitrogen fixation genes; NifB, nitrogenase molybdenum-cofactor synthesis protein; NifD, nitrogenase molybdenum-iron protein alpha chain; NifK, nitrogenase molybdenum-iron protein beta chain; NifE, nitrogenase molybdenum-cofactor synthesis protein; NoeA and NoeB, host specific nodulation proteins.

VapC10 toxin cleavage of the anticodon loop of the tRNA^Ser(GGA)^ and tRNA^Ser(UGA)^ would limit the translation of the favored codon UCC and the rare codons UCU and UCA (Fig. 3A). To characterize the role of TA toxin during symbiosis, we developed a bioinformatics approach to predict the proteins potentially impacted by VapC10 activity in nodules. This analysis was restricted to the nitrogen fixation zone (ZIII), where conditions such as oxygen limitation and acidic pH could potentially activate VapBC systems [9, 16]. We focused on the 2209 transcripts mainly expressed in the fixation zone, compared to the other zones of the nodule [49]. From them, three lists of mRNAs were defined and compared with each other, (i) the “ZIII+” list of mRNAs strongly detected in this fixation zone (1500 or more normalized reads) (ii) the “*serX/SMc02915*” list of mRNAs rich in codons decoded by tRNA^Ser(GGA)^, and (iii) the “*serT/SMc01243*” list of mRNAs rich in codons decoded by tRNA^Ser(UGA)^. The resulting Venn diagram (Fig. 3B) shows that the tRNase activity of VapC10 most likely impacts the translation of mRNAs encoding proteins essential for nitrogen fixation i.e. the nitrogenase components NifB and NifE, the transcriptional activator NifA, the oxidoreductase FixC and the ATPase FixI1. Moreover, the synthesis of the nodulation proteins NoeA and NoeB proteins, involved in host specific nodulation of particular *Medicago* spp. [50], is also potentially impacted by VapC10. The positions of the targeted serine codons in these seven transcripts are shown in Fig. S8. Even when the *serX/SMc02915* list was restricted to only mRNAs rich in the rare UCU codon (Fig. 3A), the same symbiotic mRNAs as above were identified, reinforcing our analysis (Fig. S9).

Therefore, our bioinformatics approach shows that proteins potentially impacted by VapC10 include proteins specifically involved in nodulation, nitrogen fixation, and electron transport (File S1).

To appreciate the specificity of impacted proteins, a similar approach was performed by focusing on mRNAs rich in codons translated by the tRNA^Ser(GCU)^ (*serV/SMc02409* list) and tRNA^Ser(CGA)^ (*serU/SMc03779* list) that are not targeted by VapC10 toxin (File S1). The regulator NifA, and the symbiotic proteins NifB and FixC are not present in the resulting Venn diagram (Fig. 3C), which strengthens the specific impact of VapC10 on their synthesis.

Finally, we performed a comparable bioinformatics approach to predict the number, and the replicon localization (pSymA, pSymB, or chromosome), of genes encoding proteins potentially impacted by VapC10 activity on the 6314 genes of the *S. meliloti* proteome, for the three serine codons UCC, UCU, and UCA (Table S6A, File S2). Compared to the pSymB and to the chromosome, we found that the pSymA carries more genes particularly rich in the “rare” codons UCA (“Rare SmSerT” list) and UCU (“Rare SmSerX” list). Thus, there is a bias in favor of the pSymA to carry genes rich in these codons. This difference could be the result of the origin of the pSymA, which was evolutionary acquired more recently and shows distinctive GC% and codon usage compared to the two other replicons (Table S6B).

Altogether, this *in silico* approach raises the possibility that the activity of VapC10 especially affects the translation of proteins involved in nodulation and nitrogen fixation, and thus could modulate the symbiosis efficiency.

### *vapC10*-induced nodules have an improved nitrogen fixation capacity

**Figure 4:**
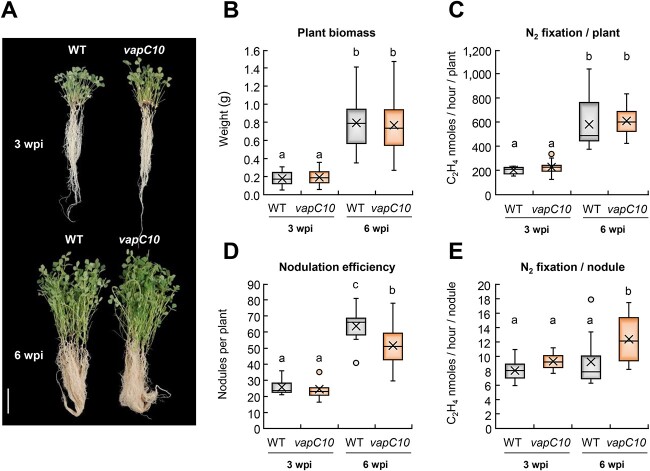
Symbiotic phenotype of *vapC10*-induced nodules; *M. truncatula* plants were inoculated with the WT or the *vapC10* mutant strains of *S. meliloti*, and the physiological symbiotic parameters were analyzed at 3- and 6- wpi; (A) phenotype of WT and *vapC10* mutant-infected plants; 15 plants inoculated with WT (left) or *vapC10* (right) strains are shown; scale bar= 5 cm; (B) weight of aerial plant part (g) (*N*=3, *n*=15); (C) nitrogenase activity per plant determined by ARA (*N*=3; *n*=5); (D) number of nodules per plant (*N*=3; *n*=5); each measure was realized on a three-plant pool and then reported by plant; (E) nitrogenase activity per nodule determined by ARA (*N*=3; *n*=5); the nomenclature (*N*=x) refers to the number of biological replicates and (*n*=x) refers to the number of measures obtained per replicate; the bars of standard deviation followed by a same letter did not differ significantly; a summary of the *P* value is shown in Table S3.

To analyze the role of the VapC10 tRNase during symbiosis, a bacterial mutant inactivated in *vapC10* toxin gene was constructed. This mutant has the same growth rate in rich medium as the WT strain, indicating no defect in free-living (Fig. S10). Then, *M. truncatula* plantlets were infected with the WT and *vapC10* mutant strains, and several physiological symbiotic parameters were analyzed at 3- and 6- wpi (Fig. 4). *M. truncatula* inoculated with the WT or the *vapC10* mutant strains had aerial plant parts of similar weight and size (Fig. 4A and B and Fig. S11), suggesting comparable nitrogen availability for plant growth. Accordingly, the nitrogen fixation capacity of plants infected by either strain was similar (Fig. 4C). This nitrogen-fixing activity, together with the number of nodules, increased between 3 and 6 wpi (Fig. 4C and D). However, at 6 wpi, the number of *vapC10* nodules per plant was significantly reduced (19% lower), compared to WT -infected nodules (Fig. 4D). Consequently, at 6 wpi, the nitrogen fixation capacity on a per nodule basis was higher in *vapC10* nodules compared to WT nodules (Fig. 4E).

Taken together, these results showed that *vapC10* mutation allows bacteria in nodule to fix nitrogen more efficiently, without impacting the general availability of nitrogen to the plant at 6 wpi.

### *vapC10* mutation delayed senescence in root nodules

**Figure 5:**
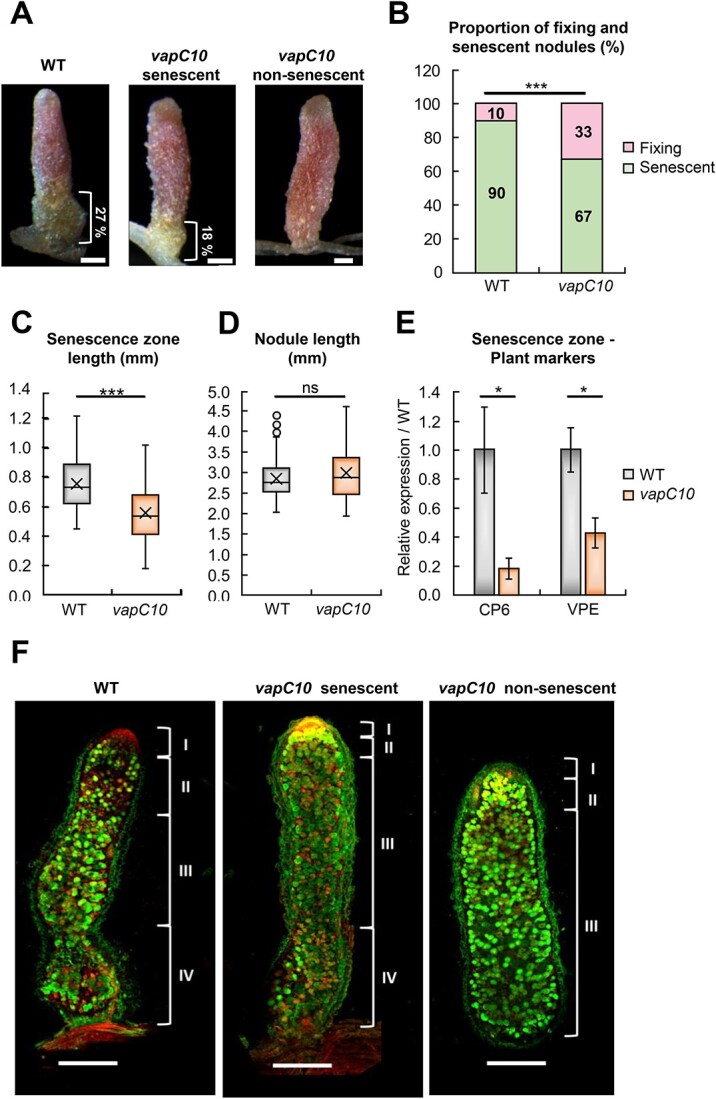
*vapC10*-induced nodules have a delayed senescence at 6 wpi; WT and *vapC10*-induced nodules were harvested at 6 wpi, on the 5 cm below the crown; (A) stereomicroscope observation of nodules; the indicated percentage corresponds to the relative size of the senescence zone compared to the nodule full length; (B) percentage of fixing (top) and senescent (bottom) nodules; (C) senescence zone length in WT and *vapC10*-induced nodules; (D) nodule size of WT and *vapC10*-induced nodules; (E) relative expression of senescence markers in *vapC10*-induced nodules by RT-qPCR: cysteine protease 6 (CP6), VPE; the gene expression is relative to the expression in the WT-induced nodules; error bars indicate the standard error of four biological replicates F; bacteroid viability in WT and *vapC10*-induced nodules; WT and *vapC10* mutant-induced nodules, semi-thin longitudinal sections (120 μm), were stained with live/dead fluorescent dyes (Live/Dead BacLight probe, Invitrogen) and observed by confocal microscopy (ZEISS LSM; objective 10×); healthy bacteroids are green, and damaged bacteroids are red; I : meristematic Zone, II : infection Zone, III: fixation Zone, IV: senescence zone; scale bars: 500 μm; observations and measures shown in (A–D) were realized on binocular magnifier (Leica MZFLIII, 0.8X) and treated on Axiovision LE software; these experiments were repeated on three biological replicates and one representative result is shown; the RT-qPCR statistical analysis was performed by a *t*-test; the representation of *P* values is as follow: ns, nonsignificant, ^*^<.05, ^*^^*^<.01, ^*^^*^^*^<.005.

The higher nitrogen fixation observed in *vapC10* nodule may be associated to a delayed nodule senescence. To test this hypothesis, macroscopic and microscopic observations of WT and *vapC10*-induced nodules were performed at 6 wpi. The nitrogen-fixing and senescence zones are easily distinguishable under the stereomicroscope. The nitrogen-fixing zone has a characteristic pink color due to the presence of functional leghemoglobin, whereas the senescence zone has a green color due to the degradation of leghemoglobin (Fig. 5A). The *vapC10* mutant induced significantly less nodules with a senescence zone (67%) compared to the WT strain (90%) (Fig. 5B, Fig. S12A). Moreover, the average length of the senescence zone was also significantly lower in *vapC10* (18% of total size) compared to WT nodules (27%) (Fig. 5A and C and Fig. S12A), although the total length of both types of nodules was similar (Fig. 5D). Altogether, these data strongly suggest that *vapC10* mutant strain induces delayed nodule senescence.

To comfort our hypothesis, the expression of *M. truncatula* gene markers associated to nodule senescence was analyzed by RT-qPCR experiments performed on RNAs extracted from nodules infected with WT or *vapC10* mutant (Fig. 5E). The cysteine protease (CP6) and vacuolar processing enzyme (VPE) genes were selected as plant markers of the senescence zone [7, 51]. A significant 5-fold and 2-fold decrease of CP6 and VPE expression, respectively, were observed in *vapC10* nodules (Fig. 5E). Lower expression of the two protease genes confirmed the delayed senescence of *vapC10* nodules. To complete our analysis, the effect of the *vapC10* mutation on the viability of bacteroids was examined by *in situ* Live/Dead staining (Fig. 5F). Living green-labelled cells were detected in the infection and nitrogen-fixation zones of all types of nodules. Only WT nodules and *vapC10* nodules with sign of senescence contained dying red labelled cells, in the basal zone of the nodules. The corresponding zone of non-senescent *vapC10* nodules was still full of living green-labelled bacteroids in the host plant-infected cells (Fig. 5F). This result was corroborated by microscopic observations using the BABB clearing technic (Fig. S12B and supplementary method), where non-senescent *vapC10* nodules harbored dense-infected cells, typical to the nitrogen-fixation zone, whereas in the senescent nodules, induced by WT or *vapC10* strains, the senescence zone was characterized by empty cells.

In conclusion, these results confirm that *vapC10*-induced nodules had a delayed senescence, thus leading to an adequate availability of nitrogen for plant growth despite a smaller number of nodules at 6 wpi, and that the absence of a functional VapC10 toxin improves the bacteroid viability. Taken together, this means that an active VapC10 toxin should negatively control the symbiotic interaction, in a WT context.

## Discussion

In this study, we identified the RNAs targeted by a TA toxin in a plant-interacting bacterium. The VapC10 toxin of *S. meliloti* was shown to target the elongator tRNA^Ser(GGA)^ and tRNA^Ser(UGA)^. To date, most of the characterized VapC toxins target the initiator tRNA, tRNA^fMet^, as demonstrated for VapCs of *S. flexneri*, *Leptospira interrogans* [52], *H. influenzae*, *S.* Typhimurium, *Bosea* sp. [53], and for VapC2 and VapC21 of *M. tuberculosis* [54]. In addition, some VapCs in *M. tuberculosis* were shown to target elongator tRNA^Gln(CUG)^ and tRNA^Leu(CAG)^ (VapC11 [55]), tRNA^Cys^ (VapC4 [56]), tRNA^Trp(CCA)^, and tRNA^Ser(UGA and CGA)^ [52]. VapC30 of *M. tuberculosis* is highly homologous to VapC10 and both toxins specifically cleave tRNA^Ser^ at similar sites in the anticodon loop, CU(U/G)G^AA, and (U/C)G^A, respectively [52]. It has been suggested that toxins targeting tRNA^fMet^ cause a strong inhibition of protein translation followed by growth arrest and bacterial cell death, whereas those targeting specific elongator tRNAs lead to a proteome reprogramming potentially involved in stress adaptation [54, 55]. For example, the activation of a TA toxin from the MazEF family (MazF-mt9) of *M. tuberculosis*, which specifically cleaves tRNA^Lys(UUU)^, leads to ribosome stalling and halt of lysine-rich protein production. This reprogramming is supposed to be essential for *M. tuberculosis* stress adaptation [57]. Likewise, the activation of the VapC4 of *M. tuberculosis* results in a proteome reprogramming involved in pathogen defense against oxidative and copper stresses [56].

In *S. meliloti*, the VapC10 toxin could also cause a partial inhibition of translation, by limiting the production of proteins containing specific serine residues. A bioinformatic approach was used to predict the proteins potentially impacted by VapC10 activity during the symbiotic interaction of *S. meliloti* with *M. truncatula*. This analysis suggests that the synthesis of key symbiotic proteins may be altered when VapC10 is functional i.e. the NifA transcriptional activator of *nif/fix* genes, the NifB and NifE components involved in the synthesis of the iron-molybdenum cofactor (FeMo-co) of the nitrogenase, the FixC oxidoreductase involved in the electron transfer to nitrogenase essential for its functioning, and the FixI1 subunit of a cation pump required for the microoxic respiration of the bacteroid. Therefore, during nitrogen-fixing symbiosis, an effective cleavage of specific tRNAs^Ser^ by VapC10 is expected to negatively affect the synthesis of proteins essential for nitrogen fixation and bacteroid survival within plant cells. Accordingly, VapC10 inactivation could improve efficiency of nitrogenase activity and viability of bacteroids. The symbiotic phenotype of the *vapC10* mutant in interaction with *M. truncatula* is consistent with this hypothesis. Indeed, at 6 wpi, this mutant induces nodules with a higher nitrogen fixation activity than nodules infected by the WT strain, and this phenotype was associated with enhanced bacteroid viability and delayed nodule senescence.

Previously, two other *vapC* mutants, *ntrR* (*vapC4*) and *vapC5*, have similarly been described to increase the nitrogen fixation efficiency in *M. sativa*, as compared to the *S. meliloti* WT strain [30-32]. As each single *vapC* mutant has a symbiotic phenotype, this suggests the lack of a functional redundancy between these three VapBC systems of *S. meliloti*. Moreover, the number of nodules induced by WT and *ntrR* or *vapC5* strains is similar, whereas *vapC10* mutant induces less nodules as compared to WT strain, which highlights a specific role for the VapC10 toxin. Altogether, the symbiotic effect of these VapC toxins could be due to different RNA targets and/or signals for triggering VapBC activation. For example, in *M. tuberculosis*, VapC-mt15 and VapC-mt11 have the same RNA targets [52] but they are specifically activated under hypoxia or macrophage infection, respectively [16]. Indeed, in human pathogens, TA toxins respond to various stresses perceived during host infection as hypoxia, low pH, oxidative stress, nutrient starvation to allow intracellular lifestyle adaptation [9, 16, 21]. The abundance of *S. meliloti* TA systems (11 VapBC among the 53 TA modules present in the bacterial genome) could be linked to the numerous plant partner signals and stress conditions encountered by *S. meliloti* during the establishment and the functioning of the fixing nodule.

During host plant interaction, *S. meliloti* must cope with various host constraints to maintain its fitness, mutualistic interaction, and nitrogen fixation capacity. Indeed, plants have adapted molecular weapons as reactive oxygen and nitrogen species, or Nodule Cysteine-Rich peptides (antimicrobial peptide equivalents) to control bacteria throughout the interaction [58-60]. In the fixation zone, growth-arrested bacteroids, supplied by plant C4-dicarboxylates, must assume nitrogen fixation reaction, costly in energy, in acidic [61], oxidizing [62], and microoxic conditions [1]. The microsymbiont is thus exposed to various micro-environments and signals that could stimulate TA activation *in planta*. For example, microoxic conditions are known to be sensed by NtrR TA toxin, because the induction level of symbiotic and metabolic genes under free-living microoxic conditions was higher in an *ntrR* mutant [63]. As proposed for NtrPR, VapBC10 module might play a role in maintaining the bacteroid metabolic balance to adapt to intracellular life in the host plant.

**Figure 6:**
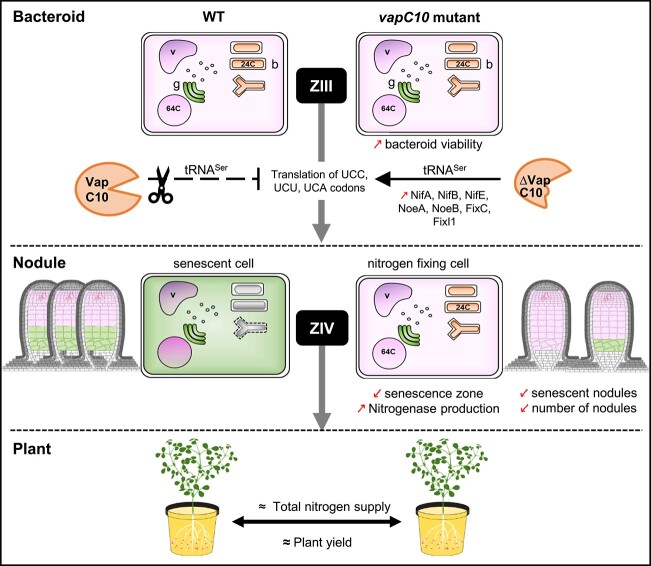
Model of VapC10 toxin role during symbiosis; schematic model summarizing the consequences of *vapC10* mutation (right panel) and deduced role of an active VapC10 toxin in a WT context (left panel) on bacteroid viability, nodule functioning, and host plant phenotype, during symbiotic interaction of *S. meliloti* with *M. truncatula*; the *vapC10* mutant induces a lower number of root nodules, but more performant in their nitrogen fixation capacity (improved bacteroid viability and delayed nodule senescence), compared to the WT strain; thus, the global nitrogen supply to the plant is satisfied, explaining a comparable plant yield obtained with the two interacting strains; in a WT context, the VapC10 toxin could act as a post-transcriptional regulator by cleaving specific tRNA^Ser^, altering the translation of symbiotic proteins involved in nitrogenase synthesis and functioning; this proteome reprogramming could limit nitrogen fixation and bacterial viability and therefore initiate bacteria and nodule senescence; the VapC10 activity could be associated to the nitrogen plant status; rectangles symbolize host plant-infected cells inside the nodules; the vacuoles (v) and the Golgi apparatus (g) are shown, the nucleus is represented by a circle containing 64 copies of the genome due to its endoreduplication (64C); bacteroids (b) are represented by rod-shaped and Y-shaped cells, the differentiated forms of bacteria (24C indicates the bacterial genome copies after endoreduplication); the pink and green colors indicate cells or nodule zones that are nitrogen fixing or senescent, respectively; ZIII: nitrogen fixation zone of the nodule; ZIV: senescence zone of the nodule; FixC, oxidoreductase regulator of nitrogenase; FixI1, ATPase; NifA, transcriptional activator of nitrogenase; NifB, FeMo cofactor biosynthesis protein; NifE, nitrogenase molybdenum-cofactor synthesis protein; NoeA and NoeB, host-specific nodulation proteins.

Our results suggest that, in a WT context, the VapBC10 TA system should limit the nodulation, the nitrogen fixation, and the bacteroid viability and should thus initiate the nodule senescence (Fig. 6). This raises the question of the benefit for the host plant and for the bacteria to limit the symbiotic interaction efficiency. Indeed, the host plant partner must satisfy a cost/benefit ratio, between the expense of photosynthetic substrates supplied to the bacteroid and the gain in nitrogen input from nitrogen fixation. This tradeoff is important for the plant to conserve energy for growth, flowering, pod filling, and possibly to cope with potential biotic and abiotic stresses. Nodulation and symbiotic fixation can therefore become more costly than beneficial to the plant if its nitrogen demand is satisfied or if assimilable nitrogen is available in the soil [64]. The *ntrR* mutant has been selected on its nodulation capacity on alfalfa, in the presence of an external source of ammonium, known to normally suppress the formation of root nodules [65]. We can propose that VapC10, and possibly VapC5 and NtrR also, sense the host plant nitrogen demand and act as post-transcriptional regulator to inhibit synthesis of proteins involved in nitrogen fixation and viability of the bacteroids. It could be particularly important to inhibit the translation of the corresponding transcripts, in a context where differentiation into bacteroids is associated to bacterial genome endoreduplication until 24C [5].

In *S. meliloti/M. truncatula* indeterminate nodules, bacteria in plant-infected cells undergo irreversible terminal bacteroid differentiation, which prevents them from escaping the nodule and returning to a free-living mode. Nodule senescence associated to bacteroid cell death, such as that induced by the VapC10 toxin, could be beneficial at the bacterial population level. Indeed, the undifferentiated bacteria from infection threads can be directly released in the rhizosphere or can grow as saprophytic bacteria in the senescent zone [66], which finally allows a significant soil enrichment in *S. meliloti*. In agreement, a recent transcriptome analysis showed that rhizobial cell cycle genes are upregulated during nodule senescence onset [67].

In conclusion, we showed that a toxin of a *S. meliloti* TA system, acting as a tRNase, limits the nitrogen fixation capacity and bacterial fitness *in planta* and thus contributes to the nodule senescent onset. VapC10 might act as a post-transcriptional regulator of symbiotic function in response to the host plant nitrogen demand (Fig. 6). It would be interesting to elucidate the symbiotic role of all other *S. meliloti* VapBC systems to determine if other *vapC* mutants also increase the nitrogen fixation capacity as found for *ntrR*, *vapC5*, and *vapC10* mutants. More generally, our study contributes to a better understanding of the biological role of bacterial TA systems in eukaryote interacting bacteria.

## Supplementary Material

File_S1_Lists_of_genes_Venn_diagrams_wrae015

File_S2_Lists_of_genes_S_meliloti_proteome_wrae015

Supplementary_information_wrae015

## Data Availability

All data generated or analysed during this study are included in this published article and its supplementary information files.
