# Supplementary material for: VapC10 toxin of the legume symbiont Sinorhizobium meliloti targets tRNASer and controls intracellular lifestyle
Source: ISME J. 2024 Jan 29;18(1):wrae015. doi: 10.1093/ismejo/wrae015 (PMC10945364; doi:10.1093/ismejo/wrae015)
Supplement: Supplementary_information_wrae015 [file supplementary_information_wrae015.pdf]

Supplementary information for:

VapC10 toxin of the legume symbiont *Sinorhizobium meliloti* targets $tRNA^{Ser}$ and controls intracellular lifestyle

Camille SYSKA, Aurélie KIERS, Corinne RANCUREL, Marc BAILLY-BECHET, Justine LIPUMA, Geneviève ALLOING, Isabelle GARCIA and Laurence DUPONT

Contents :

Figure S1. **Quality control of RNA extractions after DNase treatment measured by Bioanalyzer.**

Figure S2. **MORE RNA-seq method used to identify VapC10 toxin targets in *E. coli*.**

Figure S3. **Method used to generate lists of genes specifically expressed in the fixation zone of the nodule.**

Figure S4. **Genetic organization of the chromosomal *vapBC10* locus and VapB10 antitoxin homology to *Mycobacterium tuberculosis* VapB30.**

Figure S5. **Biological replicates of Fig. 1D and Fig. 1E.**

Figure S6. **RNA targets of VapC10 toxin identified by MORE RNA-seq, *E. coli* $tRNA^{Ser}$ homologs in *S. meliloti* and analysis of cleavage of $tRNA^{Ser}$ encoded by *serU* and *serV*.**

Figure S7. **VapC10-induced growth inhibition of *E. coli* is alleviated by expressing *S. meliloti* $tRNA^{Ser}$ targets of the toxin.**

Figure. S8. **Schematic representation of the position of the targeted serine codons UCC, UCU and UCA in the transcripts of interest.**

Figure S9. **Venn diagram of the transcripts containing specific rare serine codons in the fixation zone.**

Figure S10. **Growth kinetics of wild-type and *vapC10* strains.**

Figure S11. **Aerial plant size at 3- and 6-weeks post-inoculation**.

Figure S12. ***vapC10* induced nodules undergo delayed senescence at 6 wpi.**

Table S1. **Bacterial strains and plasmids used in this study.**

Table S2. **Primers used in this study.**

Table S3. **Summary of *p* values obtained for the symbiotic phenotype comparison of WT and *vapC10* induced nodules.**

Table S4. **Analysis of senescence in *M. truncatula* nodules induced with the wild-type or *vapC10* mutant strains at 6 wpi.**

Table S5. **Summary of sequencing read counts of each replicate obtained in the MORE RNA-seq analysis.**

Table S6. **Distribution and replicon localization of the genes potentially impacted in their translation in the *S. meliloti* proteome (A), and serine codon usage in the different replicons (B).**

Supplementary Methods

Supplementary References

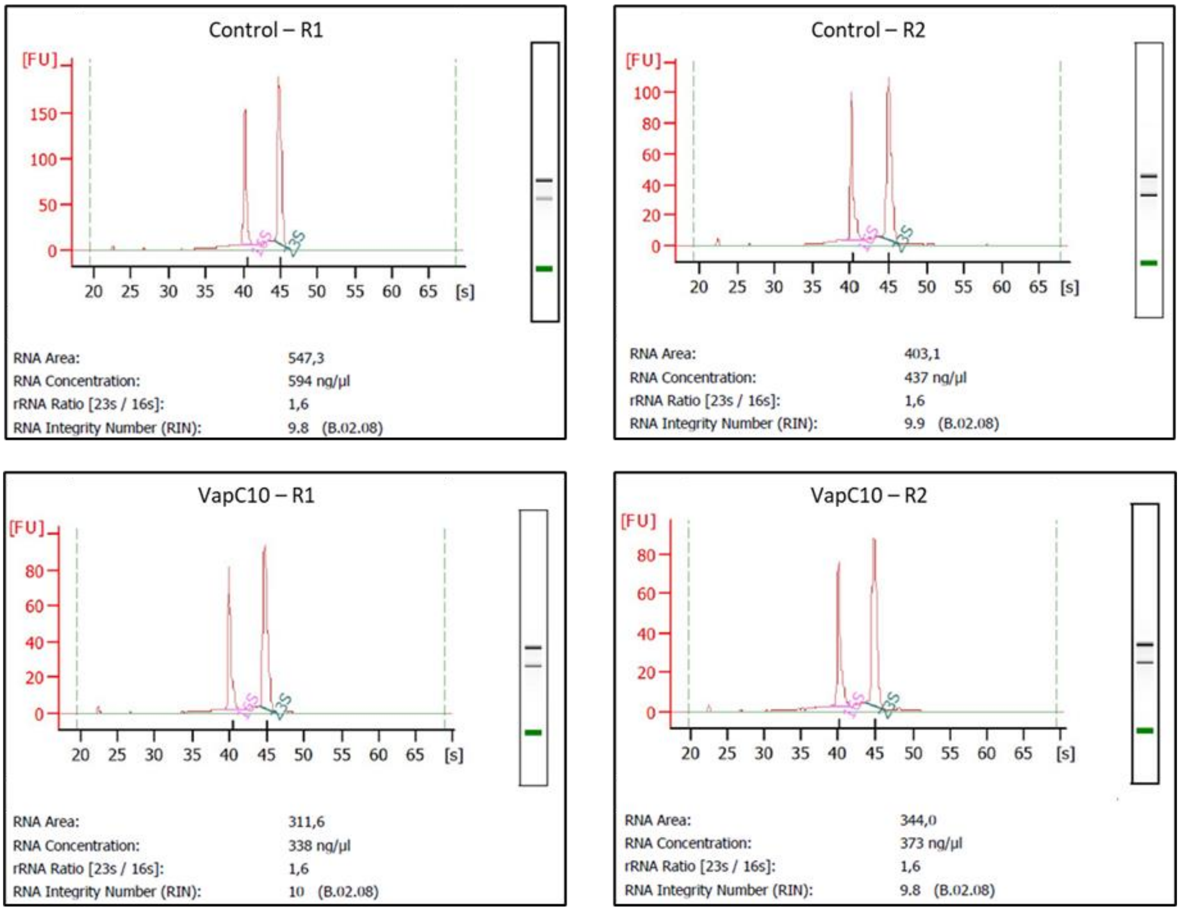

Fig. S1. Quality control of RNA extractions after DNase treatment measured by Bioanalyzer.

The results from the two biological replicates of control and VapC10 conditions are shown.

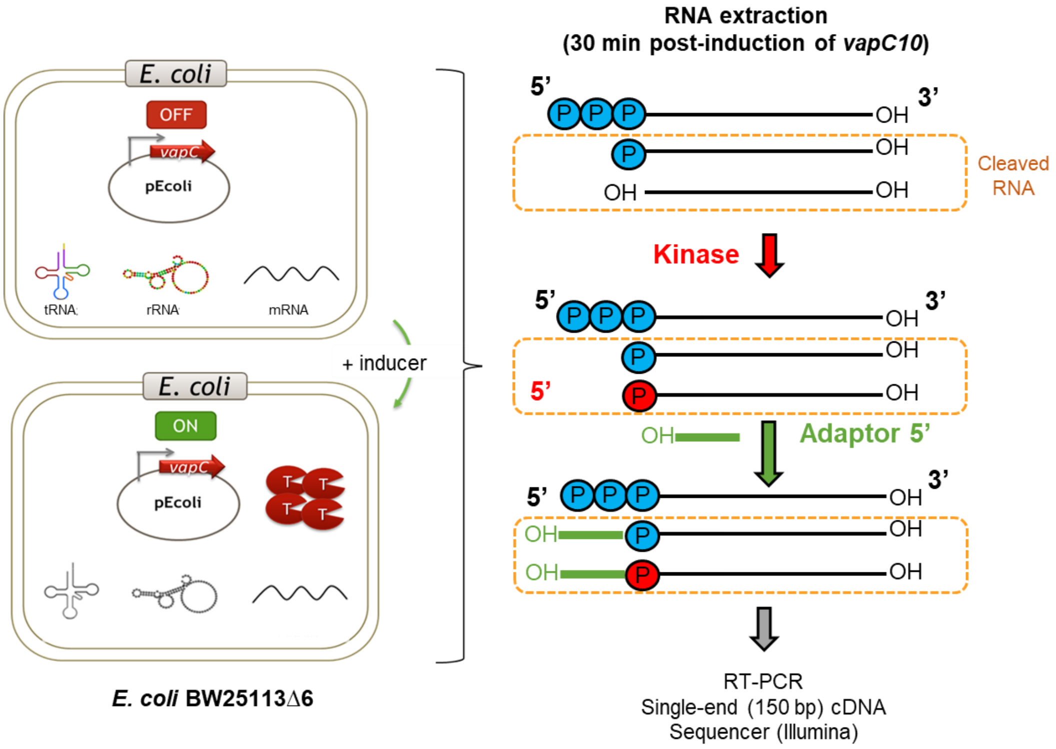

Fig. S2. MORE RNA-seq method used to identify VapC10 toxin targets in *E. coli*.

The technique used is adapted from a previously described method [ref 1]. It consists in the over-expression of a TA toxin acting as a RNase in the *E. coli* strain BW25113Δ6, deleted in six type II TA systems. VapC toxins are site-specific RNases described to primarily target tRNAs and much more rarely mRNAs or rRNAs. These three types of RNA are depicted in a bacterial cell (left). In our conditions, bacterial RNA was extracted after 30 min of induction of VapC10 toxin. As VapC10 cleavage could generate 5'-OH or 5'-monophosphate end RNAs (dotted orange rectangles), RNAs were treated with T4 polynucleotide kinase to convert 5'-OH into 5'-monophosphate ends. After ligation of the 5' and 3' adapters, the RNAs were converted to cDNAs libraries and sequenced on a MiSeq System (Illumina).

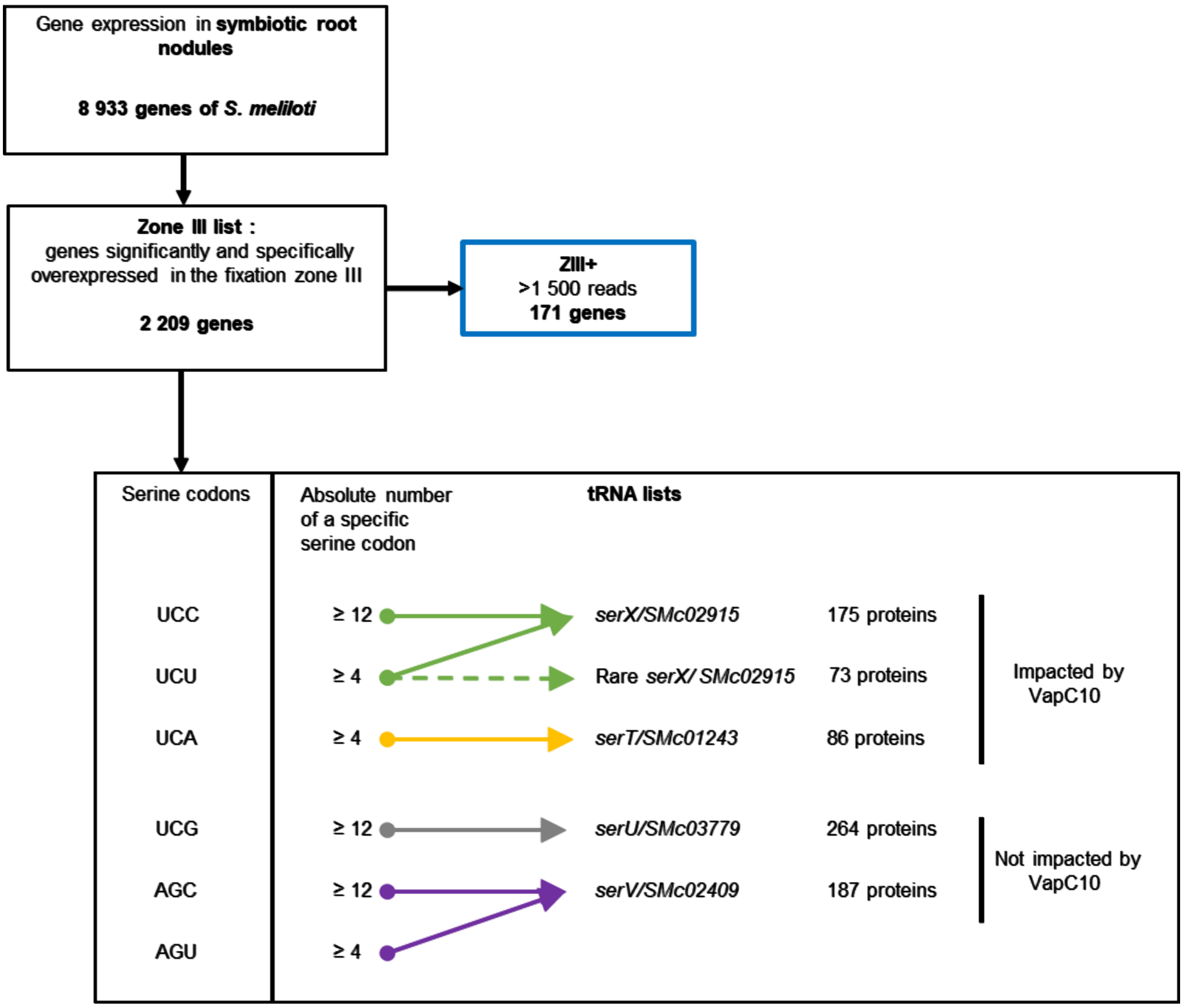

Fig. S3. Method used to generate lists of genes specifically expressed in the fixation zone of the nodule.

RNA-seq data from the laser-capture microdissection of the nodule [2] were used to select genes that are significantly and specifically overexpressed in the fixation zone (Zone III list, 2209 genes). Among them, seven lists of genes were generated: the “ZIII+” list containing the highly expressed genes (n ≥1500 reads, 171 genes), and six “tRNA lists” based on the absolute number (n) of specific serine codons in their coding sequence *i.e.*, n ≥12 (common serine codons in *S. meliloti*) and n≥4 (rare serine codons in *S. meliloti*). These six last lists are classified in “Impacted by VapC10” for genes rich in codons translated by $tRNA^{Ser}$ SMc02915 and SMc01243 and “Not impacted by VapC10” for genes rich in codons translated by $tRNA^{Ser}$ SMc03779 and SMc02409.

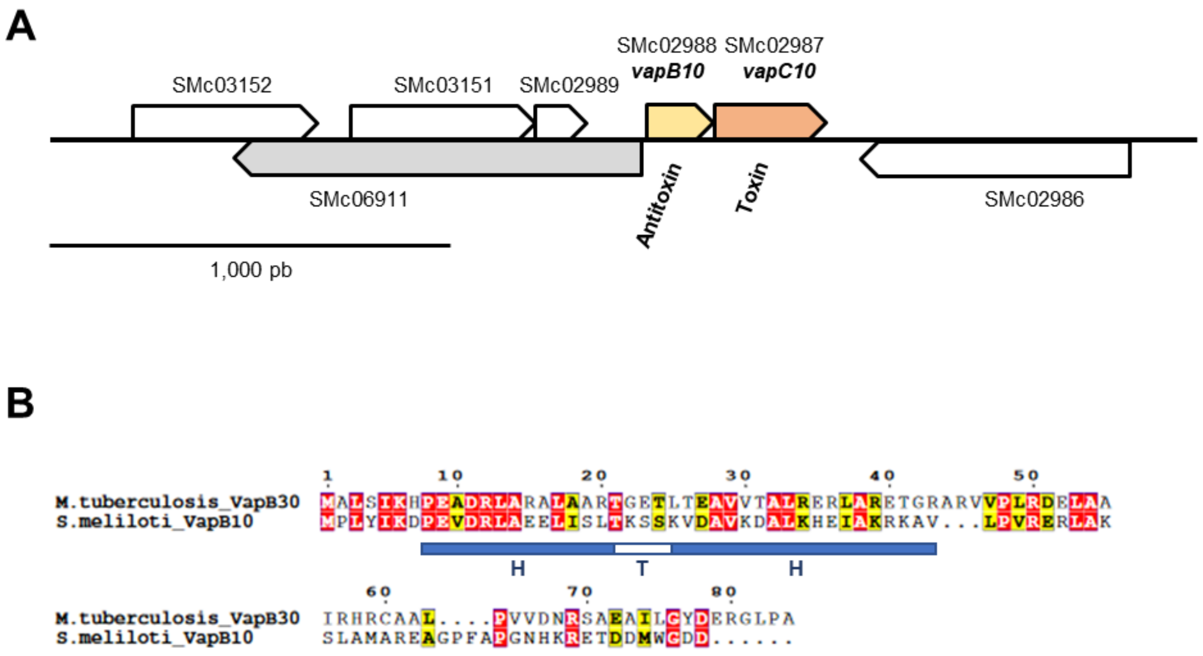

Fig. S4. Genetic organization of the chromosomal *vapBC10* locus and VapB10 antitoxin homology to *Mycobacterium tuberculosis* VapB30.

A. Genetic organization of the *vapBC10* locus. The genes represented by empty arrows encode hypothetical proteins. The SMc06911 gene specifies a ncRNA of unknown function located 18 nucleotides upstream of the *vapB10* start codon.

B. VapB10 antitoxin homology. The VapB10 protein and VapB30 antitoxin from *M. tuberculosis* (Protein Accession: P9WJ34) were aligned using PROMALS3D webserver [3], then ESPript Software was used for alignment representation. The numbers correspond to the amino acid residues. The N-terminal position of the Helix-Turn-Helix (HTH) domain is indicated by bars below the sequences. The HTH motif is deduced from InterPro 93.0 [ref 4].

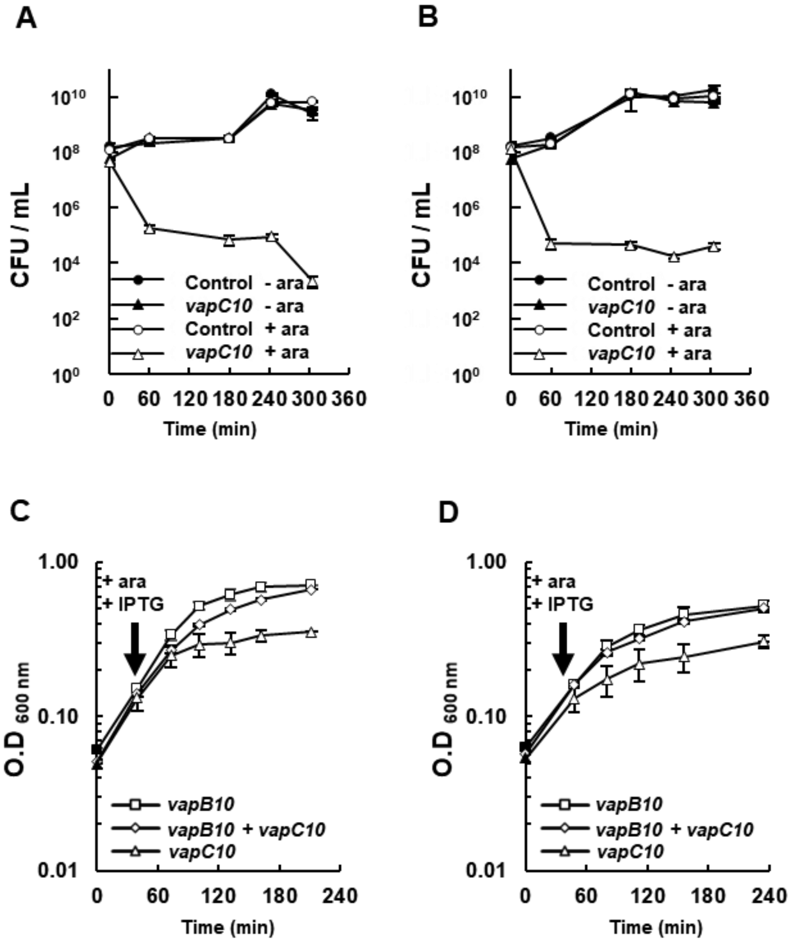

Fig. S5. Biological replicates of Fig. 1D and Fig. 1E.

(A, B) Biological replicates of the toxicity assay of the VapC10 protein (Fig. 1D). Kinetics of viability (CFU/mL) were realized in *E. coli* DH5α carrying pBAD24 (Control) or pBAD24-*vapC10* (*vapC10)* plasmids, after 1 hour of induction with 1% of arabinose (+ ara) or not (- ara).

(C-D) Biological replicates of the antitoxicity assay of VapB10 protein (Fig. 1E). Growth kinetics of *E. coli* BL21 (DE3) pLysS carrying pRSF1b-*vapB10*), pBAD24-*vapC10* or co-transformed with both plasmids. Induction by arabinose and IPTG was realized at OD_{600nm}= 0.15 (black arrow).

The means and deviations of technical duplicates from each condition are shown.

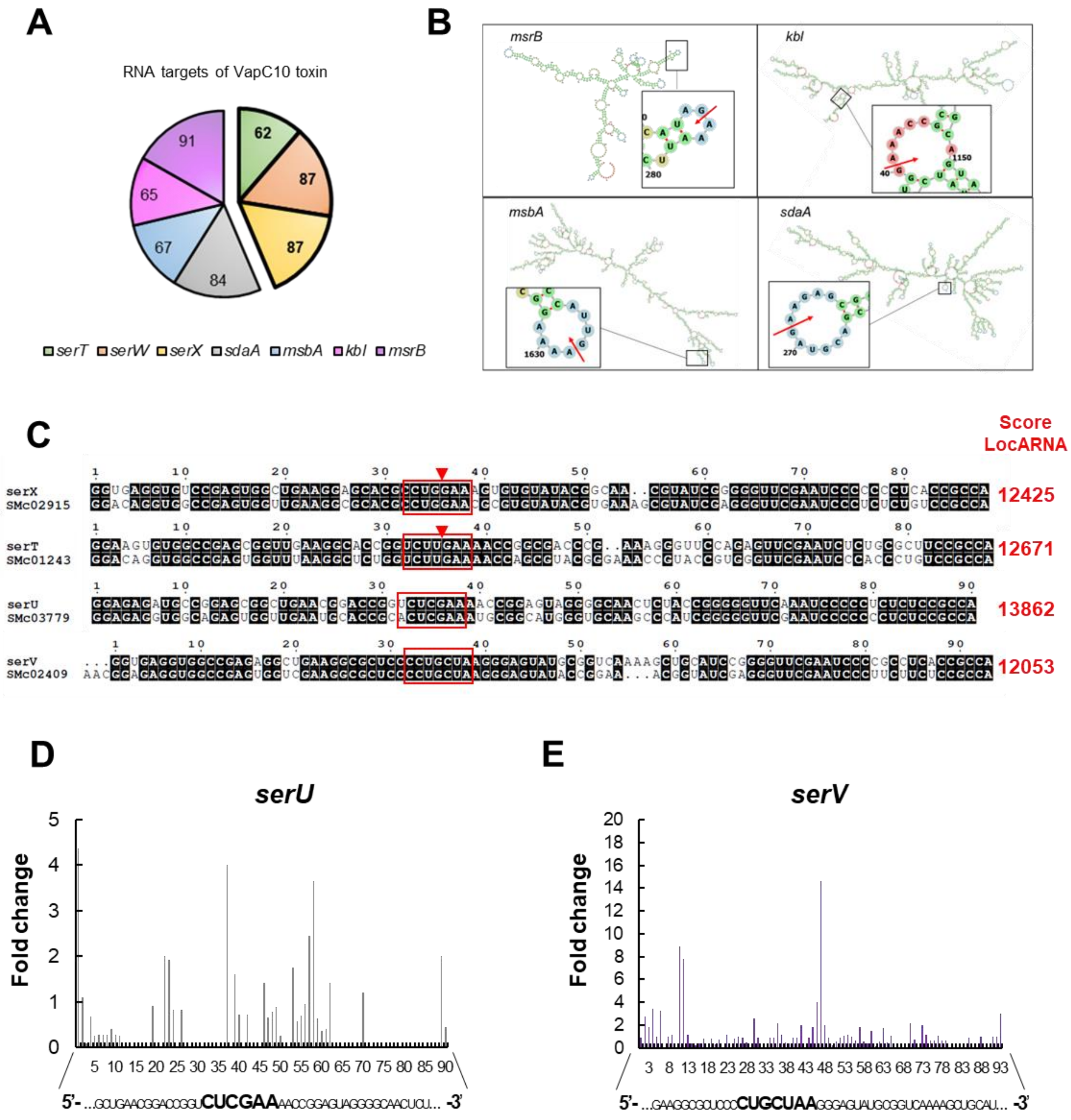

Fig. S6. RNA targets of VapC10 toxin identified by MORE RNA-seq, *E. coli* tRNA^{Ser} homologs in *S. meliloti* and analysis of cleavage of tRNA^{Ser} encoded by *serU* and *serV*.

A-B. RNA targets of the VapC10 toxin.

A. VapC10 targets seven RNAs with a cleavage ratio (enrichment of reads between the VapC10 and control conditions) greater than 60. Three correspond to tRNA^{Ser} (*serX/serW*, identical copies, and *serT*) and four to mRNA (*msrB, kbl, msbA, sdaA*). The numbers indicate the cleavage ratio.

B. Predictive secondary structure of mRNA targeted by VapC10. The secondary structure of *msrB, kbl*, *msbA* and *sdaA* was predicted with RNAFold [5]. The Minimum Free Energy structure was visualized using forRNA tool [6]. The red arrow indicates the VapC10 cleavage site, the numbers correspond to nucleotide positions and the colors are related to the structure (green: stem, blue: hairpin loop, red: multiloop-junction, yellow: interior loop).

msrB: methionine sulfoxide reductase B; *kbl*: glycine C-acetyltransferase; *msbA*: lipid A–core ATPase transporter; *sdaA*: L-serine dehydratase.

C. LocARNA alignment of $tRNA^{Ser}$ from *E. coli* and *S. meliloti*. The LocARNA score is indicated on the right of the alignments [7]. The red box corresponds to the anticodon loop sequence, and the red arrow to the cleavage site of VapC10.

D-E. The $tRNA^{Ser}$ specified by *serU* and *serV* are not cleaved by VapC10 toxin. Histograms representing the ratio of sequencing reads (Fold change) between the induced and non-induced VapC10 conditions, in the $tRNA^{Ser(UCG)}$ (*serU*) **(D)** and in the $tRNA^{Ser(GCU)}$ (*serV*) **(E)**, at each nucleotide position. The anticodon is shown in bold letters in the tRNA sequences. The numbers shown below the histograms indicate the nucleotide positions of *serU* and *serV* tRNAs.

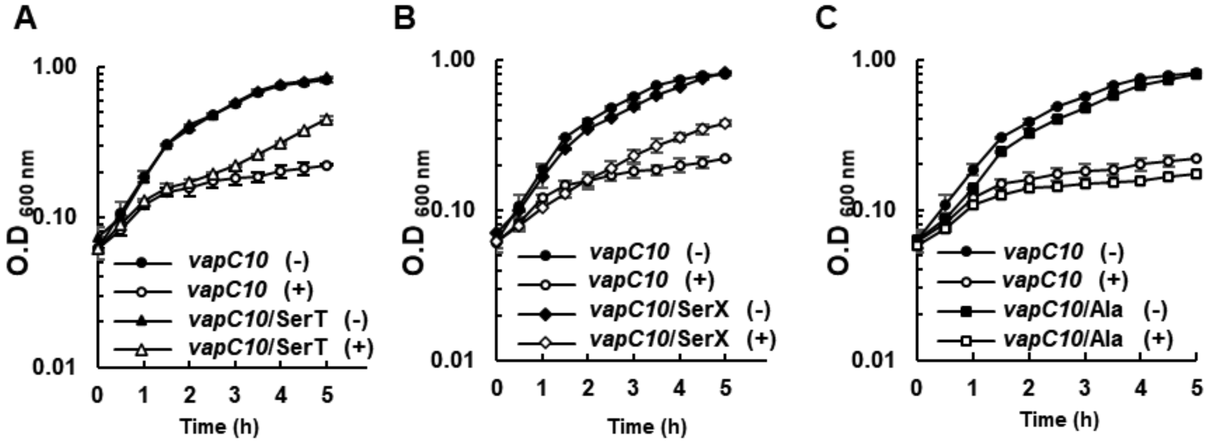

Fig. S7. VapC10-induced growth inhibition of *E. coli* is alleviated by expressing *S. meliloti* tRNA^{Ser} targets of the toxin.

E. coli cultures carrying pBAD24-*vapC10* alone (*vapC10*) or together with (A) pSmSerT (*vapC10/*SerT), (B) pSmSerX (*vapC10*/SerX), or (C) pSmAla (*vapC10*/Ala), were grown overnight under non-inducing conditions, then diluted to an OD_{600nm} of 0.05 and further grown in the presence of both arabinose and IPTG inducers (+), or not (-).

Means of two independent cultures are shown. Results are representative of at least two biological replicates.

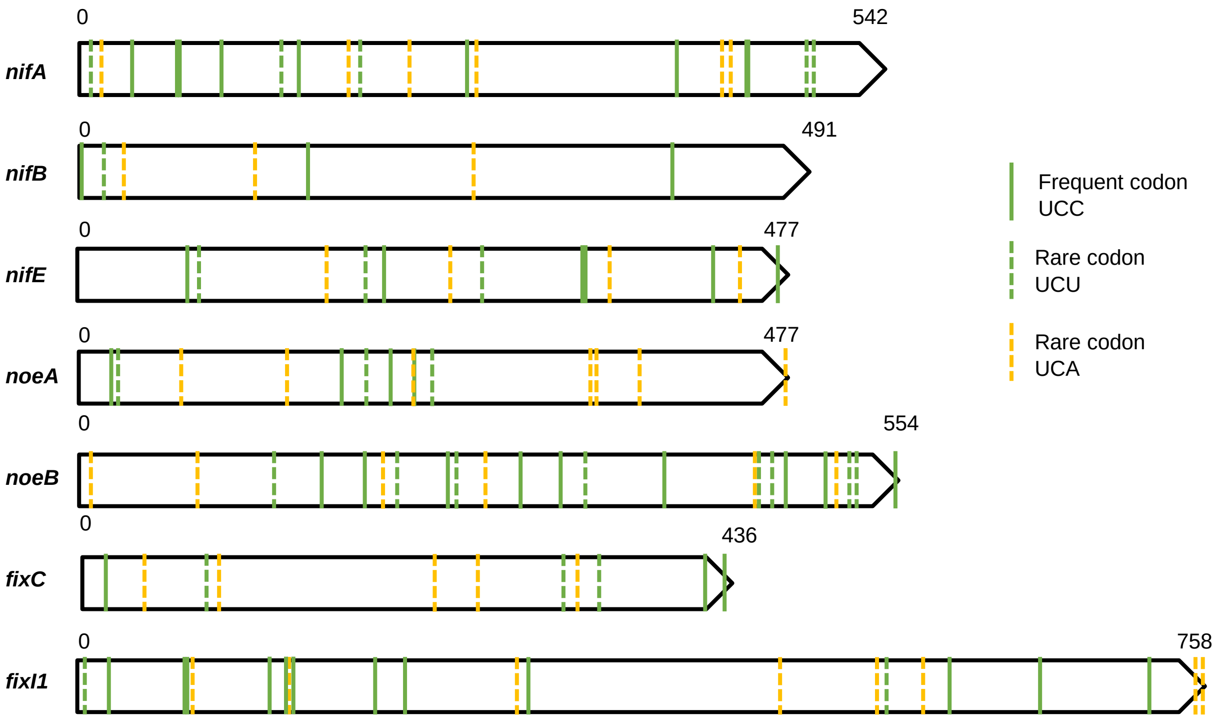

Fig. S8. Schematic representation of the position of the targeted serine codons UCC, UCU and UCA in the transcripts of interest.

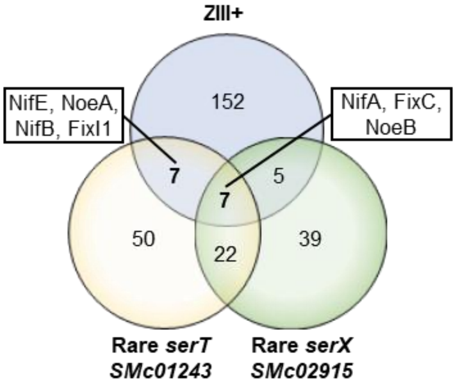

Fig. S9. Venn diagram of the transcripts containing specific rare serine codons in the fixation zone.

Transcripts rich in rare serine codons translated by tRNA$^{Ser(UGA)}$ and tRNA$^{Ser(GGA)}$, potentially impacted by VapC10 cleavage. A Venn diagram was used to identify genes expressing mRNAs containing at least 4 rare UCA codon (named Rare *serT/SMc01243* list), or rare UCU codon (named Rare *serX/SMc02915* list), among the specifically and highly expressed genes of the fixation zone III (named ZIII+ list).

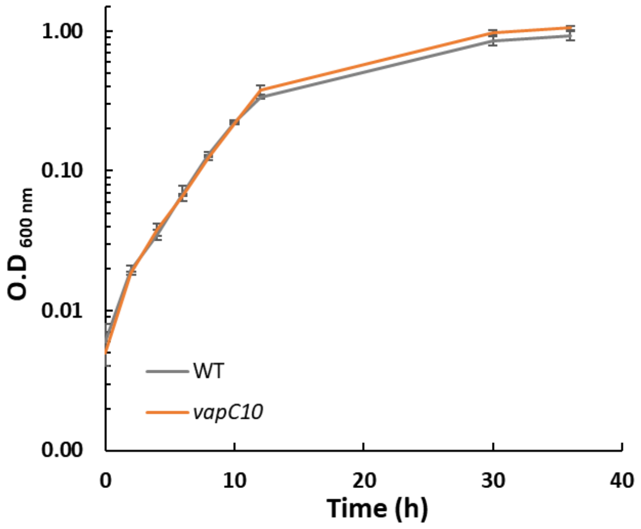

Fig. S10. Growth kinetics of wild-type and *vapC10* strains.

The cultures of the wild-type (WT) and *vapC10* mutant strains were performed in rich medium (LB_{MC}) at 30°C and the growth was followed by OD_{600nm} measurements. Results are representative of three independent biological replicates. The means and deviations of the technical duplicates from each condition are shown.

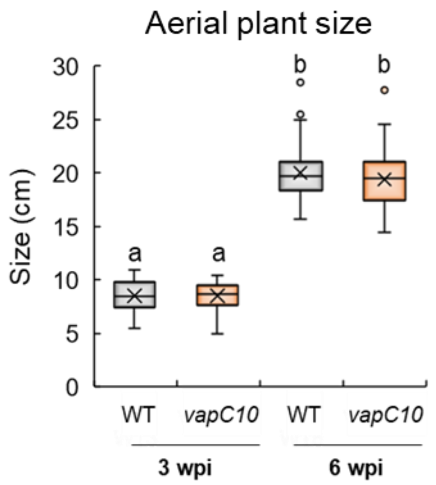

Fig. S11. Aerial plant size at 3- and 6-weeks post-inoculation.

M. truncatula plants were inoculated with the wild-type or the *vapC10* mutant strains of *S. meliloti*, and the aerial plant size (cm) was measured at 3- and 6-weeks post-inoculation (wpi), (N=3, n=15). Each measurement was realized on a three-plants pool and then reported by plant.

The nomenclature (N=x) refers to the number of biological replicates and (n=x) refers to the number of measurements per replicate. The significance of differences was estimated by one-way analysis of variance (ANOVA). A post hoc Tukey's test was applied for pairwise comparisons. The means with the same letter did not differ significantly.

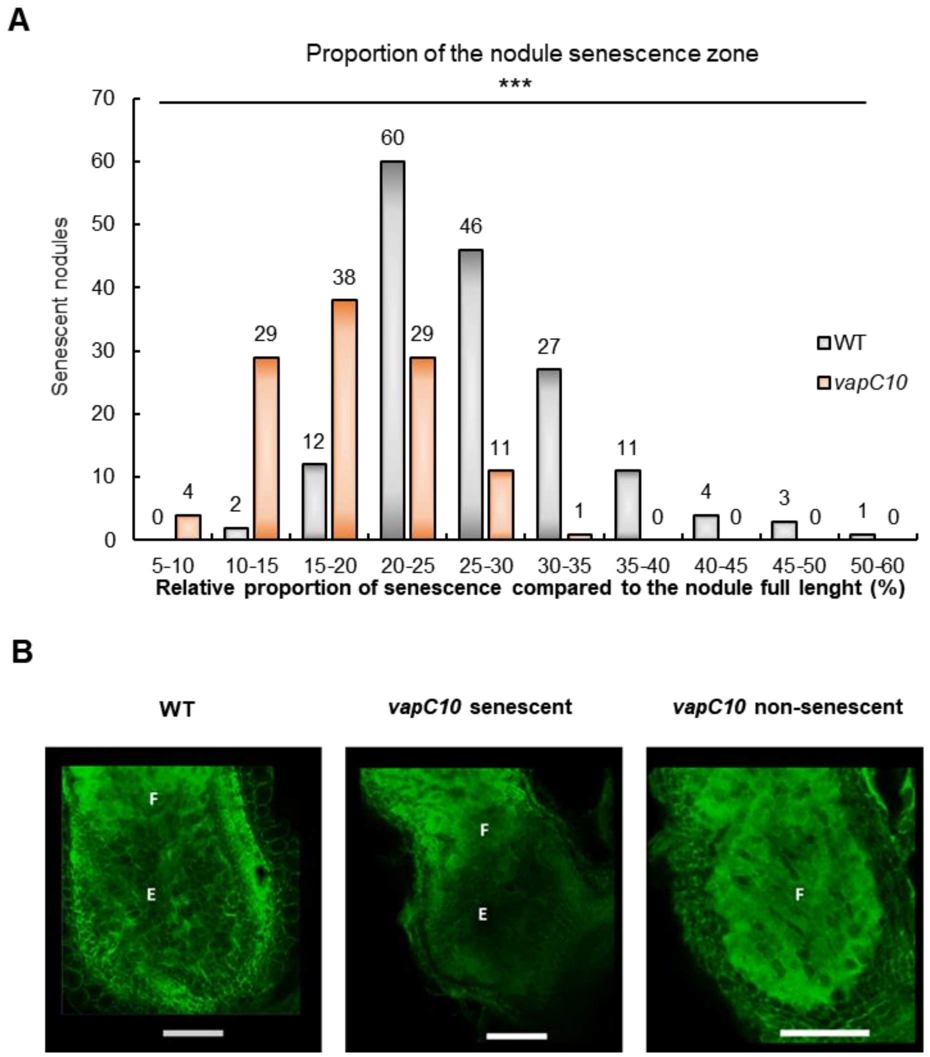

Fig. S12. *vapC10* induced nodules undergo delayed senescence at 6 wpi.

Wild-type (WT) and *vapC10* induced nodules were harvested at 6 wpi, within the first 5 cm down the crown and their senescence were analyzed by microscopy.

A. Distribution of WT and *vapC10* induced nodules according to their senescence status. Fixing and senescent zones were distinguished based on their color (pink: fixing zone, green: senescent zone) using a stereomicroscope. The percentages correspond to the relative size of the senescent zone compared to the nodule full length. The representation of *p* value is as follow: ***<0.005.

B. Microscopic observation of wild-type and *vapC10*-induced nodules cleared by BABB technique. Observations were realized on ZEISS LSM confocal microscope (objective 10x). Images correspond to representative examples on three biological replicates. Scale bars: 200 µm. E: empty senescent cells, F: fixing cells.

Strains	Description	References
Escherichia coli		
DH5α	*endA1 glnV44 thi-1 recA1 relA1 gyrA96 deoR nupG purB20 φ80dlacZΔM15 Δ(lacZYA-argF)U169, hsdR17(rK–mK+)*	[8]
BL21 (DE3) pLysS	*ompT gal dcm lon hsdSB(rB–mB–) λ(DE3 [lacI lacUV5-T7p07 ind1 sam7 nin5]) [malB+]K-12(λS) pLysS[T7p20 orip15A],* Cm^R	Novagen
BW25113Δ6	*[lacIq rrnBT14 Δlac-ZWJ16 hsdR514 ΔaraBADAH33 ΔrhaBADLD78 ΔchpBIK ΔdinJ-yafQ ΔhipBA ΔmazEF ΔrelBE ΔyefM-yoeB]*	[1, 9]
Sinorhizobium meliloti		
Sm2011	SU47 derivative, referred to wild-type or Sm2011, Sm^R	[10]
vapC10	Sm2011 derivative, containing pKmob19Ω2HMB plasmid insertion within *vapC10* gene, referred to as *vapC10* mutant, Sm^R Neo^R	This work
**Plasmids**	**Description**	**References**
Cloning plasmid construction		
pJET1.2	Cloning vector, Amp^R	ThermoFisher
pJET-*vapC10-Sm*	pJET1.2 derivative, containing a fragment of *vapC10* gene (from 7th to 255th nucleotide position, on a total of 444 nucleotides)	This work
pJET-*vapC10-tox*	pJET1.2 derivative, containing *vapC10* gene	This work
pJET-*vapB10-tox*	pJET1.2 derivative, containing *vapB10* gene	This work
S. meliloti mutant construction		
pK19mob2ΩHMB	Suicide vector, Kan^R/Neo^R	[11]
pK19mob2ΩHMB-*vapC10*	pK19mob2ΩHMB derivative, containing a fragment of *vapC10* gene (from 7th to 255th nucleotide position, on a total of 444 nucleotides)	This work
Expression of vapC10 toxin and vapB10 antitoxin in E. coli		
pBAD24	Expression vector, arabinose inducible promoter (pBAD), colE1 ori, Amp^R	[12]
pBAD24-*vapC10*	pBAD24 derivative, containing *vapC10* gene	This work
pRSF1b	Expression vector, IPTG inducible promoter (T7), RSF 1030 ori, Kan^R	Novagen
pRSF1b-*vapB10*	pRSF1b derivative, containing vapB10 gene	This work
Expression of S. meliloti tRNAs in E. coli		
pRSF1bΔHE	pRSF1b derivative, with a HpaI-Eco53KI deletion	This work
pSmSerT	pRSF1bΔHE, containing the *S. meliloti* tRNA SMc01243 homologous to tRNA-SerT *of E. coli*	This work
pSmSerX	pRSF1bΔHE, containing the *S. meliloti* tRNA SMc02915 homologous to tRNA-SerX *of E. coli*	This work
pSmAla	pRSF1bΔHE, containing the *S. meliloti* tRNA Ala SMc01244	This work

Table S1. Bacterial strains and plasmids used in this study.

Gene	Gene ID	Primer name	Primer sequence	Cloning vector
Primers used for the vapC10 mutant construction				
vapC10	SMc02987	VapC10-HindIII-pk19-F	5'-TGC**AaGCTt**TTCATTGACGCCTCGGTTAT-3'	pK19mob2ΩHMB
		VapC10-BsrGI-pk19-R	5'-C**tgtacA**ATCATCGCTTCCTTGCAATC-3'	
Primers used for toxicity and antitoxicity assays				
vapB10	SMc02988	VapB10-BsaI-Tox-F	5'-CCTCGAAAGGA**ggTctcGc**ATG-3'	pRSF1b
		VapB10-XhoI-Tox-R	5'-GAATGGC**ctCGAg**AACCGAGG-3'	
vapC10	SMc02987	VapC10-BsaI-Tox-F	5'-TGTGGGGC**GgTctCTc**ATGCTGTTCATTG-3'	pBAD24
		VapC10-SalI-Tox-R	5'-CAGGGTTTGGC**GTcGac**TTCCGATAGACC-3'	
Primers used for the cloning of S. meliloti tRNAs				
tRNA^{Ser}	SMc01243	SMc01243-F	5'-AT**CTGCAG**TAATACGACTCACTATAGGGACAGGTGGCCGAGTG-3'	pRSF1bΔHE
		SMc01243-R	5'-AT**CTCGAG**CCCGCCAACAATGCCAC-3'	
tRNA^{Ser}	SMc02915	SMc02915-F	5'-AT**CTGCAG**TAATACGACTCACTATAGGGACAGGTGGCCGAGTG-3'	pRSF1bΔHE
		SMc02915-R	5'-AT**CTCGAG**GCGAATAAAGGACTAACTC-3'	
tRNA^{Ala}	SMc01244	SMc01244-F	5'-AT**CTGCAG**TAATACGACTCACTATAGGGGGTCGTAGCTCAGTTG-3'	pRSF1bΔHE
		SMc01243-R	5'-AT**CTCGAG**GCTTTTTTGGCAAGCAGC-3'	

Gene	Gene ID	Description	Primer sequence	Ref
Primers used for RTqPCR of plant gene normalization				
Mtc27	MtrunA17_Chr2g0295871	Housekeeping gene	F : 5'-GTGGGAGGTTGAGGGAAAGT-3'<br>R : 5'-TTGAAGGTCCTTGAGCTTGC-3'	[13]
A38	MtrunA17_Chr4g0061551	Housekeeping gene	F : 5'-TCGTGGTGGTGGTTATCAAA-3'<br>R : 5'-TTCAGACCTTCCCATTGACA-3'	[13]
Primers used for RTqPCR of plant gene expression				
MtVPE	MtrunA17_Chr1g0151801	Vacuolar Processing Enzyme	F : 5'-CCAGGGGTTCTTGGTATGCCCG-3'<br>R : 5'-ACTGCCAGATTCACATGCCTCCA-3'	[14]
MtCP6	MtrunA17_Chr4g0041081	Cysteine Protease	F : 5'-CCTGCTGCTACTATTGCTGGATATG-3'<br>R : 5'-CACTCGCATCAATGGCTACGG-3'	[14]

Table S2. Primers used in this study.

The restriction sites are shown in bold with mismatches in lower cases. The T7 promoter sequence in primers used for *S. meliloti* tRNA genes is underlined.

	N_2 fixation / nodule		N_2 fixation / plant		Plant weight		Number of nodules per plant	
**Compared conditions**	*p value adjusted*	*Significance*	*p value adjusted*	*Significance*	*p value adjusted*	*Significance*	*p value adjusted*	*Significance*
vapC10 6 wpi -*vapC10* 3 wpi	1.40E-02	*	1.70E-09	***	8.09E-14	***	2.28E-09	***
WT 3 wpi-*vapC10* 3 wpi	5.55E-01	ns	9.64E-01	ns	9.87E-01	ns	9.84E-01	ns
WT 6 wpi -*vapC10* 3 wpi	1.00E+00	ns	3.46E-08	***	8.09E-14	***	0.00E+00	***
WT 3 wpi -*vapC10* 6 wpi	2.41E-04	***	3.17E-10	***	8.09E-14	***	8.11E-09	***
WT 6 wpi -*vapC10* 6 wpi	1.30E-02	*	9.41E-01	ns	9.19E-01	ns	7.26E-03	**
WT 6 wpi -WT 3 wpi	6.29E-01	ns	6.75E-09	***	8.09E-14	***	0.00E+00	***

Table S3. Summary of *p* values obtained for the symbiotic phenotype comparison of WT and *vapC10* induced nodules.

For cross-analysis between WT and *vapC10* infected plants, at 3- or 6-wpi, a Shapiro test was used to control the normality of the values and a Bartlett test for the homogeneity of variances. One-way analysis of variance (ANOVA) followed by the Tukey post-hoc test was used for testing equality of means in N_2 fixation experiments, weight of the aerial parts of plants and for the number of nodules. The representation of *p* values is as follow: ns, non-significant, *<0.05, **<0.01, ***<0.005.

Parameters analyzed	Wild-type	*vapC10*	Test	*p* value
**Total number of nodules**	185	167	NA	NA
**Number of non-senescent nodules**	19	55	Binomial	9.89E-08 ***
**Number of senescent nodules**	166	112		
**Percentage of senescent nodules**	89.7%	67.1%		
**Average length of nodule (mm)**	2.85 ± 0.46	2.99 ± 0.62	Wilcoxon	0.07 ns
**Average length of fixation zone (mm)**	2.08 ± 0.33	2.53 ± 0.45	Wilcoxon	4.46E-11 ***
**Average length of senescence zone (mm)**	0.75 ± 0.14	0.56 ± 0.15	Wilcoxon	1.89E-14 ***
**Average percentage of senescence in senescent nodules**	27.2% ± 6.8	18.2% ± 5.0	Wilcoxon	2.2E-16 ***

Table S4. Analysis of senescence in *M. truncatula* nodules induced with the wild-type or *vapC10* mutant strains at 6 wpi.

Wild-type and *vapC10* induced nodules were harvested at 6 wpi, within the first 5 cm down the crown. The count of senescent or non-senescent nodules, and the estimation of fixing and senescence zones were based on macroscopic observation of their color (pink: fixing zone, green: senescence zone). The indicated percentages correspond to the relative proportion of senescent nodules compared to the total number of nodules, and to the relative size of the senescence zone compared to the nodule full length.

The observations were made on binocular magnifier (Leica MZFLIII, 0.8X) and analyzed on Axiovision LE software. Data correspond to three biological replicates.

The significance of differences between the proportions of senescent nodules in WT and *vapC10* induced nodules was tested by a Chi-squared test. The significance of differences between i) the lengths of nodules, fixation and senescence zones in WT and *vapC10* induced nodules, and ii) the average percentage of senescence in senescent nodules induced by WT and *vapC10* strains, was tested by a Wilcoxon test. The representation of *p* values is as follow: ns, no significant, *<0.05, **<0.01, ***<0.005.

Biological replicate	***Condition***	***Total raw reads***	***Total trimmed reads***	***Total mapped reads***	***rRNA %***	***non rRNA %***
R1	pBAD24	18,768,230	9,326,821	7,554,725	71.3	28.7
	pBAD24-*vapC10*	20,727,253	10,548,784	8,781,863	71.6	28.4
R2	pBAD24	19,231,192	10,959,367	9,614,653	76.5	23.5
	pBAD24-*vapC10*	15,285,300	9,244,037	8,321,482	77.1	22.9

Table S5. Summary of sequencing read counts of each replicate obtained in the MORE RNA-seq analysis.

R1 and R2 indicates the two biological replicates.

A

Lists	All	pSymA		pSymB		Chromosome	
	Number of genes	Number of genes	Percentage (%)	Number of genes	Percentage (%)	Number of genes	Percentage (%)
**Proteome**	6314	1341	21.25	1585	25.11	3386	53.64
**Sup12 UCC "Common SmSerX"**	320	42	13.21	72	22.64	204	64.15
**Sup4 UCU "Rare SmSerX"**	162	71	43.83	38	23.46	53	32.72
**Sup4 UCA "Rare SmSerT"**	185	106	57.30	41	22.16	38	20.54

B

	Serine codons cleaved by VapC10		
	Common SmSerX	Rare SmSerX	Rare SmSerT
**Codon Usage %**	**UCC**	**UCU**	**UCA**
**pSymA**	22.01	**6.95**	**8.10**
**pSymB**	24.45	**4.79**	**4.87**
**Chromosome**	26.59	**4.18**	**3.92**

Table S6. Distribution and replicon localization of the genes potentially impacted in their translation in the *S. meliloti* proteome (A), and serine codon usage in the different replicons (B).

Supplementary methods:

Supplementary Methods Figure S6: The VapC10-induced growth inhibition of *E. coli* is alleviated by expressing *S. meliloti* tRNA^{Ser} targets of the toxin.

Expression of tRNAs of *S. meliloti* in *E. coli*

To demonstrate that the tRNAs SMc01243 and SMc02915 are specifically cleaved by VapC10, we performed a genetic approach in *E. coli*, based on the strategy used by Walling and Butler [16]. Thus *SMc01243, SMc02915* and tRNA^{Ala}-encoding *SMc01244,* were expressed in *E. coli* on pRSF1bΔHE, a pRSF1b derivative. In this plasmid, the region of pRSF1b encompassing the T7 promoter and lac operator was deleted (Table S1). Using appropriate primers (Table S2), DNA fragments containing the T7 promoter directly upstream of SMc01243, SMc02915 or SMc01244 ORFs were first amplified by PCR from genomic DNA of *S. meliloti* strain 2011. PCR fragments were thereafter cloned into the pJET1.2 vector, then sub-cloned into the PstI/XhoI sites of pRSF1bΔHE, leading to pSmSerT, pSmSerX and pSmAla, respectively (Table S1). These plasmids were finally introduced into the BL21 (DE3) pLysS strain of *E. coli* containing the pBAD24-*vapC10* plasmid. Growth kinetics were performed in the absence or presence of arabinose (1%) and IPTG (0.1 mM) to induce the expression of VapC10 and tRNA, respectively.

Supplementary Methods Figure S9: The *vapC10*-induced nodules undergo delayed senescence at 6 wpi.

Nodule histology using BABB clearing

Histological comparison of the wild-type and *vapC10* induced nodules was performed using the BABB clearing technique as described previously[15] with minor modifications. Briefly, as 6 wpi-old nodules are thick, the last clearing step in BABB solution (benzyl alcohol /benzyl benzoate mixed in a 1:2 proportion (*v*/*v*)) was maintained one week at 4°C. Acquisition of images was performed using a Zeiss confocal laser scanning microscope (LSM880).

Supplementary references:

1. Schifano JM, Vvedenskaya IO, Knoblauch JG, Ouyang M, Nickels BE, Woychik NA. An RNA-seq method for defining endoribonuclease cleavage specificity identifies dual rRNA substrates for toxin MazF-mt3. Nat Commun. 2014; https://doi.org/10.1038/ncomms4538.
2. Roux B, Rodde N, Jardinaud M-F, Timmers T, Sauviac L, Cottret L, et al. An integrated analysis of plant and bacterial gene expression in symbiotic root nodules using laser-capture microdissection coupled to RNA sequencing. Plant J. 2014; 77:817-837.
3. Pei J, Kim BH, Grishin N V. PROMALS3D: A tool for multiple protein sequence and structure alignments. Nucleic Acids Res. 2008; 36:2295-2300.
4. Paysan-Lafosse T, Blum M, Chuguransky S, Grego T, Pinto BL, Salazar GA, et al. InterPro in 2022. Nucleic Acids Res. 2023; 51:D418-D427.
5. Gruber AR, Lorenz R, Bernhart SH, Neuböck R, Hofacker IL. The Vienna RNA websuite. Nucleic Acids Res. 2008; 36:W70-W74.
6. Kerpedjiev P, Hammer S, Hofacker IL. Forna (force-directed RNA): simple and effective online RNA secondary structure diagrams. Bioinformatics. 2015; 31:3377-3379.
7. Will S, Joshi T, Hofacker IL, Stadler PF, Backofen R. LocARNA-P: accurate boundary prediction and improved detection of structural RNAs. RNA. 2012; 18:900-914.
8. Sambrook J, Fritsch EF, Maniatis T. Molecular cloning: a laboratory manual (2nd ed.). Cold Spring Harbor, NY: Cold Spring Harbor Laboratory Press, 1989.
9. Prysak MH, Mozdzierz CJ, Cook AM, Zhu L, Zhang Y, Inouye M, et al. Bacterial toxin YafQ is an endoribonuclease that associates with the ribosome and blocks translation elongation through sequence-specific and frame-dependent mRNA cleavage. Mol Microbiol. 2009; 71:1071-1087.
10. Rosenberg C, Boistard P, Dénarié J, Casse-Delbart F. Genes controlling early and late functions in symbiosis are located on a megaplasmid in *Rhizobium meliloti*. Mol Gen Genet. 1981; 184:326-333.
11. Luo L, Yao S-Y, Becker A, Rüberg S, Yu G-Q, Zhu J-B, et al. Two new *Sinorhizobium meliloti* LysR-type transcriptional regulators required for nodulation. J Bacteriol. 2005; 187:4562-4572.

12. Guzman LM, Belin D, Carson MJ, Beckwith J. Tight regulation, modulation, and high-level expression by vectors containing the arabinose P_{BAD} promoter. J Bacteriol. 1995; 177:4121-4130.

13. del Giudice J, Cam Y, Damiani I, Fung-Chat F, Meilhoc E, Bruand C, et al. Nitric oxide is required for an optimal establishment of the *Medicago truncatula-Sinorhizobium meliloti* symbiosis. New Phytol. 2011; 191:405-417.

14. Pierre O, Hopkins J, Combier M, Baldacci F, Engler G, Brouquisse R, et al. Involvement of papain and legumain proteinase in the senescence process of *Medicago truncatula* nodules. New Phytol. 2014; 202:849-863.

15. Cabrera J, Olmo R, Ruiz-Ferrer V, Abreu I, Hermans C, Martinez-Argudo I, et al. A phenotyping method of giant cells from root-knot nematode feeding sites by confocal microscopy highlights a role for *CHITINASE-LIKE 1* in *Arabidopsis*. Int J Mol Sci. 2018; https://doi.org/10.3390/ijms19020429.

16. Walling LR, Butler JS. Homologous VapC toxins inhibit translation and cell growth by sequence-specific cleavage of $tRNA^{fMet}$. J Bacteriol. 2019; 200:e00582-17.
